# Quantitative assessment of collagen architecture from routine histopathological images shows concordance with Second Harmonic Generation microscopy

**DOI:** 10.1371/journal.pdig.0001364

**Published:** 2026-10-26

**Authors:** Vedant Ingawale, Krishnendu Dandapat, Jyothsna Konkada Manattayil, Shashank Gupta, L. S. Shashidhara, Chaitanyanand Koppiker, Nameeta Shah, Varun Raghunathan, Madhura Kulkarni

**Affiliations:** 1 Centre for Translational Cancer Research, Indian Institute of Science Education and Research, Pune, and Prashanti Cancer Care Mission, Pune, India; 2 Prashanti Cancer Care Mission, Pune, India; 3 Ashoka University, Sonipat, India; 4 Department of Electrical Communication Engineering (ECE), Indian Institute of Science, Bangalore, India; 5 Amaranth Medical Analytics, Bangalore, India; 6 National Center for Biological Sciences, Bangalore, India; University of Chicago Pritzker School of Medicine, UNITED STATES OF AMERICA

## Abstract

Collagen organization within the tumour microenvironment plays a critical role in tumour progression and has emerged as an important structural biomarker in cancer. Second Harmonic Generation (SHG) microscopy enables label-free visualization and quantitative assessment of fibrillar collagen architecture; however, its high cost, specialized instrumentation, and limited field-of-view restrict routine clinical application. In this study, we evaluated whether collagen features quantified from digitally scanned Masson–Goldner’s Trichrome-stained histopathological sections can approximate measurements obtained from SHG microscopy. Formalin-fixed paraffin-embedded breast tumour tissues, including benign and invasive ductal carcinoma (IDC) samples with varying collagen content, were analysed using SHG microscopy and whole-slide brightfield imaging. Matched regions of interest were analysed using two independent digital image analysis approaches: a conventional ImageJ-based workflow (TWOMBLI) and a machine learning–based computational pipeline. Collagen structural parameters including collagen deposition area, fibre number, and alignment metrics were quantified and compared across imaging modalities using correlation analysis. SHG signals were consistently detected from Trichrome-stained sections, confirming compatibility of SHG imaging. Quantitative comparison demonstrated significant concordance between SHG-derived collagen metrics and those obtained from digital image analysis pipelines, particularly for collagen area and fibre alignment. These findings demonstrate a proof-of-concept, that computational analysis of routine histopathological images can capture key spatial features of collagen organization comparable to SHG microscopy. Digital pathology–based collagen quantification therefore represents a potentially scalable and clinically accessible approach for assessing extracellular matrix architecture in tumour tissues.

## Author summary

Quantitative assessment of extracellular matrix architecture remains a critical yet underutilized dimension of cancer pathology. While Second Harmonic Generation (SHG) microscopy provides a gold-standard, label-free approach to interrogate fibrillar collagen organization, its limited accessibility has prevented integration into routine clinical workflows. This creates a translational gap between advanced imaging capabilities and potential scalable clinical implementation. Here, we address this gap by demonstrating that collagen structural features derived from routine histopathological whole-slide images can approximate SHG-derived measurements. Using matched regions from breast tumour tissues, we perform a systematic cross-modality comparison between SHG microscopy and two independent computational pipelines, a conventional ImageJ-based workflow and a machine learning–based framework. We observe strong concordance across key spatial metrics, including collagen deposition, fibre density, and alignment. Importantly, we establish that SHG imaging is fully compatible with Trichrome-stained sections, enabling direct validation on identical tissue regions. Our findings show that widely available digital pathology data, when coupled with computational analysis, can recover biologically and clinically relevant features of collagen architecture previously accessible primarily through specialized optical systems. This work introduces a potentially scalable, cost-effective, and clinically translatable framework for extracellular matrix quantification, with implications for biomarker discovery, computational pathology, and integration of stromal features into digital health platforms.

## Introduction

Second Harmonic Generation (SHG) signals arising from the highly polarizable, ordered, non-centrosymmetric fibrillar structure of collagen were first reported nearly four decades ago [1]. Since then, SHG imaging has emerged as a powerful optical approach for investigating collagen organization and deposition in biological tissues [2,3]. Because SHG selectively detects fibrillar collagen without the need for exogenous labelling, it enables clear distinction between fibrillar collagen and surrounding non-fibrillar tissue components. This capability has enabled quantitative assessment of collagen deposition and architecture in several disease contexts. For example, SHG has been used to quantify fibrillar collagen deposition in liver fibrosis for prognostic evaluation [4,5].

In cancer biology, SHG imaging has revealed clinically relevant features of the extracellular matrix. In breast cancer, SHG-derived Tumour Associated Collagen Signatures (TACS) have been identified as independent prognostic indicators of disease-free survival [6,7]. Similarly, in pancreatic ductal adenocarcinoma, SHG-derived collagen deposition scores have been shown to correlate with overall patient survival [8]. These studies highlight the importance of collagen organization and deposition as clinically informative features of the tumour microenvironment.

Despite these advantages, the application of SHG microscopy has largely remained confined to research settings. The high cost of instrumentation, requirement for specialized expertise, relatively limited field of view, and the time required for tissue imaging pose practical barriers to its implementation in routine diagnostic workflows [9]. In contrast, collagen-specific histochemical stains such as Masson’s Trichrome, Picrosirius Red, and immunohistochemical detection using collagen-specific antibodies are routinely used in clinical pathology to visualize and report collagen deposition and stromal architecture. With the increasing adoption of digital pathology, these stained tissue sections can now be digitized at high resolution and computationally analysed to extract quantitative features of collagen organization.

Recent studies have therefore begun exploring whether collagen features quantified from digitized histopathological images of stained tissue sections can compare to the information obtained from SHG microscopy, thereby offering a more accessible route for collagen analysis in clinical settings. However, systematic comparisons between SHG-derived collagen measurements and those obtained from routinely stained histopathological sections remain limited.

In this study, we aim to provide a proof-of-concept. We investigate whether collagen features quantified from digital images of Masson–Goldner’s Trichrome-histopathological sections are comparable to those derived from SHG imaging. Because SHG-based collagen quantification is conventionally performed on unstained tissue sections, with only limited reports on stained sections, we first evaluated the compatibility of SHG microscopy for collagen fibre detection in Trichrome-stained slides to determine whether Trichrome staining interferes with SHG-based collagen detection. The SHG signals obtained from Trichrome-stained sections were compared with those from corresponding unstained and haematoxylin and eosin (H&E) stained tissue sections.

Following validation of SHG compatibility with Trichrome-stained sections, the same slides were digitized using whole-slide imaging. Matched regions of interest (ROIs) corresponding to the SHG-imaged areas were identified and subjected to quantitative collagen analysis using two independent image analysis approaches: a conventional ImageJ-based workflow and a machine learning (ML)-based computational pipeline.

In summary, our study demonstrates that collagen structural features quantified from digitally scanned Masson-Goldner’s Trichrome-stained histopathological sections show strong concordance with measurements obtained from SHG microscopy. We further show that SHG imaging is compatible with Trichrome-stained tissue sections, enabling direct comparison between the two imaging modalities on the same tissue regions. Computational analysis of whole-slide digital images using both conventional image analysis and machine learning–based pipelines was able to capture detailed collagen fibre features and spatial patterns comparable to those derived from SHG imaging. Importantly, the digital pathology-based approach offers practical advantages including larger field-of-view, compatibility with routine clinical staining protocols, and potential scalability for high-throughput analysis. Together, these findings suggest that quantitative analysis of collagen features from routinely acquired histopathological images may provide a feasible and clinically accessible alternative for assessing collagen organization in tumour tissues.

Through this comparative analysis aimed to evaluate methodological feasibility, we find that collagen features derived from routine histopathological imaging can approximate those obtained through SHG microscopy and evaluate the potential of digital pathology–based approaches as scalable and clinically implementable alternatives for quantitative collagen analysis in tumour tissues.

## Methods

### Patient tissue samples and ethics statement

Primary breast tumour tissue (Formalin-fixed paraffin embedded, FFPE) was received from the biobank [10], with formal written patient consent and ethical approval. Ethical approval was given for the project titled “Molecular profiling of Indian Triple-Negative Breast Cancer”, on September 30^th^, 2022, by a registered Independent Ethics Committee (Registration EC/NEW/INST/2021/2443 Dated 12/May/2022). Two IDC tumour samples, one with low collagen and another with high collagen deposition, and one benign tumour sample were selected for the study purpose. To minimise staining batch effects, all samples were processed in a single batch.

### Sectioning of FFPE tumour blocks

FFPE blocks of the selected samples were processed for histopathology. Three sections of 3–5 μm were obtained from each selected tumour sample using Leica Microtome RM2255.

### Processing of unstained sections

One deparaffinised and rehydrated tumour tissue section of each sample was gradually dehydrated in ethanol solutions followed by Xylene. Slides were then mounted in Dibutylphthalate Polystyrene Xylene (DPX, catalogue number: Q18404).

### H&E staining

One tissue section of each sample was deparaffinized and gradually rehydrated with ethanol solutions further each slide was cleaned with distilled water and stained with a drop of undiluted Haematoxylin solution (Delafield, 38,803) in a humidifying chamber for 15 min. Slides were then rinsed and subjected to an acid-alcohol wash and immediately transferred to a running water bath 15 mins. This was followed by 1% eosin (catalogue number: Qualigen Q39312) staining for 30 secs. The slides were then gradually dehydrated in ethanol solutions followed by Xylene. Slides were mounted in DPX (catalogue number: Q18404).

### Trichrome staining

For Masson Goldner’s Trichrome staining, deparaffinised and rehydrated section of each sample was incubated in Bouin’s solution at 56°C for one hour. Following a wash, these were stained with prepared Weigert’s iron haematoxylin (Haematoxylin - Sigma-Aldrich, catalogue number: H3136-25G) for ten minutes at room temperature and then kept in running water for 5 mins. Slides were then sequentially stained with a drop of Azophloxine (Millipore, catalogue number: 273742) for 7 mins, Tungstophosphoric Acid Orange G solution (Millipore, catalogue number: 273744) for 6 mins, and Light Green SF (Millipore, catalogue number: 273745) for 8 mins. These were interspersed with a 30 sec 1% acetic acid wash. The slides were then gradually dehydrated in ethanol solutions followed by Xylene. Slides were mounted in DPX (catalogue number: Q18404).

### WSI acquisition of stained slides

Whole Slide Imaging (WSI) was done for each slide imaged with an objective magnification of 40x and resolution of 0.253 μm/pixel by OptraScan using OS-15 bright field digital scanner. Images were checked for focus and quality, converted to TIFF format. WSI TIFF files were uploaded to PathPresenter. Regions of interest for SHG imaging were annotated by a trained researcher (MK) to capture representative areas of tissue morphology.

### Experimental setup for SHG and TPEF image acquisition of stained and unstained slides

Subsequently directed to a harmonic generator (APE HarmoniXX SHG) to generate a harmonic wavelength at 800 nm, which was used as the source of excitation to acquire second harmonic generation (SHG) and two-photon excitation fluorescence (TPEF). The sample was mounted in an inverted position on a x-y stage of the inverted optical microscope (Olympus IX73). A water immersion objective (60x/1.2 NA) was used to focus the input laser onto the sample to collect the backward emitted SHG signal at 400 nm. The SHG and TPEF images from the breast tissue samples for same field of view was collected separately using a photomultiplier tube (Hamamatsu R3896) with two different filter sets of wavelength range 400 ± 40 nm and 520 ± 40 nm, respectively. The incident power at the focus for the tissue samples was 1.75 mW. The sample images are acquired by scanning the laser beam with a pair of galvanometric mirrors (Thorlabs GVS002). In the first round of imaging, three 100 × 100 µm^2^ FOVs were captured referencing the ROI annotations for the IDC High Collagen sample to specifically validate the SHG signal and assess compatibility across different staining protocols (S1 Fig). Once confirmed that SHG microscopy is compatible with reading Trichrome-stained slides to visualise collagen fibres, we carried out a second round of imaging to capture a larger tissue area to visualise and quantify macroscopic collagen architecture. Digitally scanned images span across the entire tissue sample, whereas SHG image acquisition of such a large region would require a significantly large amount of time. Therefore, in the second round of imaging, 25 images each of size 103 × 103 µm^2^ (234 × 234 pixels^2^) with an overlap region of 11 µm were acquired and stitched to generate a ROI of size 470 × 470 µm^2^ compromising on time taken to image while attempting to capture as larger tissue region possible. The pixel size was calculated from a US Airforce (USAF) calibration of the SHG microscope, based on elements 1, 3, 4, and 6 of Group 5 (S2 Fig). The average pixel size from this calibration comes out to be 0.44 µm/pixel (S1 Data, Sheet 1). The diffraction limited optical resolution is ~ 300nm. Two such ROIs were imaged for each tissue sample, namely benign tissue, IDC tissue with low collagen deposition and IDC tissue with high collagen deposition.

### SHG image analysis

The SHG images from Trichrome stained slides were analysed using ImageJ to measure collagen deposition patterns. Analysis was done for twenty-five FOVs of 103 µm × 103 µm dimension each sample as well as the stitched ROIs of 470 µm × 470 µm dimension. The acquired SHG images were processed in MATLAB and ImageJ to extract key quantitative parameters including mean SHG signal, and fibre width. Built-in MATLAB image processing filters were applied to suppress noise, and histogram equalization was used to enhance the contrast of the raw images. Frangi filter was applied onto the contrast enhanced image to sharpen the edges of the fibres. To ensure strict separation between fibre and background, binarization was done on the contrast enhanced image with and without frangi filter using Otsu’s method resulting in white fibres against a black background [11]. The resulting binary images were combined using logical ‘OR operation’ to retrieve all the fibril pixels representing the complete fibril map. The combined mask was subsequently multiplied with the raw image to isolate fibril-associated intensities, enabling quantification of the mean SHG signal. This signal, indicative of the structural organization and ordering of collagen within the field of view, was computed as the average SHG intensity over pixels corresponding strictly to the segmented fibrils. As background pixels are completely excluded by the mask, the average intensity provides an unbiased metric that is positively correlated with collagen fibrillar density [12]. In parallel, the binary mask was applied to the contrast-enhanced image to extract fibril structures for morphological analysis. The extracted fibres from the contrast-enhanced images were further analysed using a ridge detection algorithm in ImageJ to estimate the fibre width.

For the calculation of collagen area, SHG images underwent denoising, contrast enhancement and binarization. The percentage of collagen area was measured from these binary images, by calculating the ratio of collagen-positive pixels with respect to the total pixel area of the image. The pre-processed images were also used as inputs for the widely used TWOMBLI macro on ImageJ to get endpoints, branchpoints and coherence measurements [13]. Orientation frequencies of collagen fibres from 470 µm ROIs were measured using ImageJ’s OrientationJ plugin [14] with a local window of 2 pixels, cubic spline gradient and minimum energy 10%.

### Digital image analysis with TWOMBLI

ROIs corresponding to the SHG images were extracted from the digital brightfield image of the Trichrome stained WSI using ImageScope software by aligning identifiable objects in the image (S1 Fig). These ROIs were 1716 × 1716 pixels with a resolution of 0.253 µm, thus with a height and width of 435 µm that corresponded to 470 µm dimensions of an SHG ROI. The digital image ROIs were then deconvoluted in QuPath [15] and further binarized using auto thresholding in ImageJ, area was measured. Binarized images were skeletonised using the TWOMBLI macro on ImageJ, a widely used tool specifically developed to quantify extracellular matrix patterns as shown in Fig 5A. Quantification of endpoints, branchpoints, coherence, and box count was done using this macro. Separately, the 435 µm ROIs were further cropped into 25 FOVs of 87 µm in height and width for each ROI, to correspond to individual FOV images on SHG. These 25 FOVs per image were also analysed using TWOMBLI analysis pipeline. For getting one to one concordance of FOVs between SHG images and digital images, the direction of snake sorting numbering was flipped on the digital image, as digital ROIs were mirror images of SHG ROIs. Number of fibres were calculated as described in Wershof *et al.*, 2021, estimated as half the sum of the number of endpoints (E) and branchpoints (B) computed by the TWOMBLI macro [13]. Alignment of fibres with respect to each other is represented by the coherency metric, yielding values in the range of 0–1, with zero representing isotropy while one reflecting perfect alignment. The complexity of collagen deposition pattern was represented as box count, ranging from 1 to 2, where one represents a one-dimensional fractal, and two represents more complex fractals filling the space of a two-dimensional plane. The values lesser than 1 represent geometrical dust like object, spaces with finite disconnected points [16]. The spatial parameters of collagen fibres analysed from the 87 µm patches were plotted for each tissue sample. Orientation frequencies of collagen fibres from 435 µm ROIs were measured using ImageJ’s OrientationJ plugin [14] with a local window of 10 pixels, cubic spline gradient and minimum energy 10%.

### Digital image analysis with ML

ML analysis employs a sophisticated python-based multi-step approach to isolate collagen and can be found on GitHub (https://github.com/Amaranth-Medical-Analytics/trichrome-collagen). The pipeline combines colour-based clustering with a pre-trained U-net model to extract collagen fibres from Masson’s Trichrome-stained images as shown in Fig 5F. To enable use of the U-net model originally developed and trained specifically for SHG imaging, the brightfield images were computationally transformed into an ‘SHG-like’ representation through domain adaptation. The U-Net has been developed by Park and colleagues in 2023 [17]. This process starts with colour normalisation, where each RGB value was clipped to background values and rescaled to minimise staining variability across slides. The green channel was subsequently selected as the primary surrogate for collagen signal. To extract the initial collagen regions Gaussian smoothing was applied to reduce noise, followed by Mini-Batch K-Means clustering to group pixels based on their RGB colour profiles, utilising a subsample of up to 40,000 pixels for efficient fitting with the number of clusters set to 50. A dynamic background detection logic then identifies collagen clusters by iteratively identifying background clusters using high-intensity thresholds. The remaining clusters were classified as collagen based on the green-channel dominance and non-grey colour variations, ensuring that even faint fibres are accurately captured while minimizing false positives from non-fibrous areas.

Rigorous artifact suppression was performed to reduce noise from cellular and non-collagenous structures. A residual tissue mask identifying non-collagen components was dilated using a small elliptical kernel. This conservative morphological expansion accounted for boundary uncertainties and these expanded regions were subtracted from the initial collagen mask to improve structural specificity. The final SHG-like input was generated by masking the normalised green channel with this refined collagen mask, followed by Gaussian smoothening to better match the appearance of SHG images. These images were then processed using a convolutional neural network (CNN)-based U-Net model. This architecture provided a robust framework for segmenting fine collagen fibres by leveraging high-resolution patch-level inputs.

Following collagen detection and segmentation, the extracted fibres were analysed to compute several key metrics characterizing their structural and morphological properties. Circularity statistics were calculated to evaluate the angular orientation of fibres, including the mean circular angle (cir_mean) and its variance (cir_var), which represented the directional consistency of the fibres. Area statistics comprised number of pixels positive for collagen, alongside the total number of fibres (num_fib) detected and length of each fibre. Together, these metrics provided a comprehensive statistical framework for characterizing collagen architecture and its spatial organization.

### Comparison of collagen parameters quantified from two different modalities with three analysis pipelines

Spatial parameter outputs for collagen deposition area, fibre count, Fibre orientation were calculated from both imaging modalities for 25 FOVs from 2 ROIs from each tumour sample type. The outputs from SHG image analysis, brightfield image analysis with Image J pipeline and brightfield image analysis with ML analysis pipeline were compared with each other for concordance using Spearman Correlation analysis. Histograms of orientation frequency distribution from SHG and digital images for each ROI were plotted together comparison.

### Statistical analysis

All statistical tests were performed using GraphPad Prism version 8.0.1 for Windows with a significance threshold (alpha) at 0.05. Non-parametric tests were performed since data was not normally distributed. For comparison of means of quantified collagen parameters across ROIs of the three types of samples Kruskal-Wallis test was performed along with Dunn’s multiple comparison test for multiple comparisons. Multiplicity corrected p values were reported for Dunn’s multiple comparison test. Correlation between quantified parameters from different image types and analysis methods was checked using two-tailed Spearman Correlation.

## Results

### SHG signal detection from three distinctly processed tissue sections

To verify the presence of collagen fibres and to check whether staining protocols interfere with nonlinear optical imaging we assessed the compatibility of SHG microscopy across unstained, H&E-stained and Masson-Goldner’s Trichrome-stained tissue sections. Through the same non-linear optical microscope setup, three different ROIs of 100 µm were imaged for SHG and TPEF across the three slide preparations for an IDC tumour section with known high collagen deposition.(Fig 1A). The unstained slide represents a baseline control as it is the conventional standard used in SHG imaging for collagen fibre detection. A strong SHG signal was observed from the unstained slide indicating presence of collagen fibres with high degree of alignment, though an optical reference for this slide is inherently unavailable (Fig 1B). A TPEF signal was also observed from both extracellular and cellular features; however, the magnitude of this signal was lesser than that of the SHG signal (Fig 1B). In the combined image, the overlap of SHG and TPEF highlights the fibrillar collagen in the context of cellular structures. For the H&E slide, SHG and TPEF signal were of comparable magnitude indicating the presence of collagen fibres along with the eosin-stained cellular features, as seen in the optical reference images (Fig 1C). Notably, the SHG signal from the Trichrome stained slide (Fig 1D) showed the highest magnitude compared to that of unstained and H&E slides. Across all preparations TPEF signal was generally an order of magnitude lower than the SHG signal, except in the H&E sample. Hence, SHG signal images, and not TPEF images were used for further analysis of collagen fibre characterisation. The signal output from the SHG channel was consistent across the three slides, with the signal from the Trichrome stained slide being the highest in magnitude.

**Fig 1:**
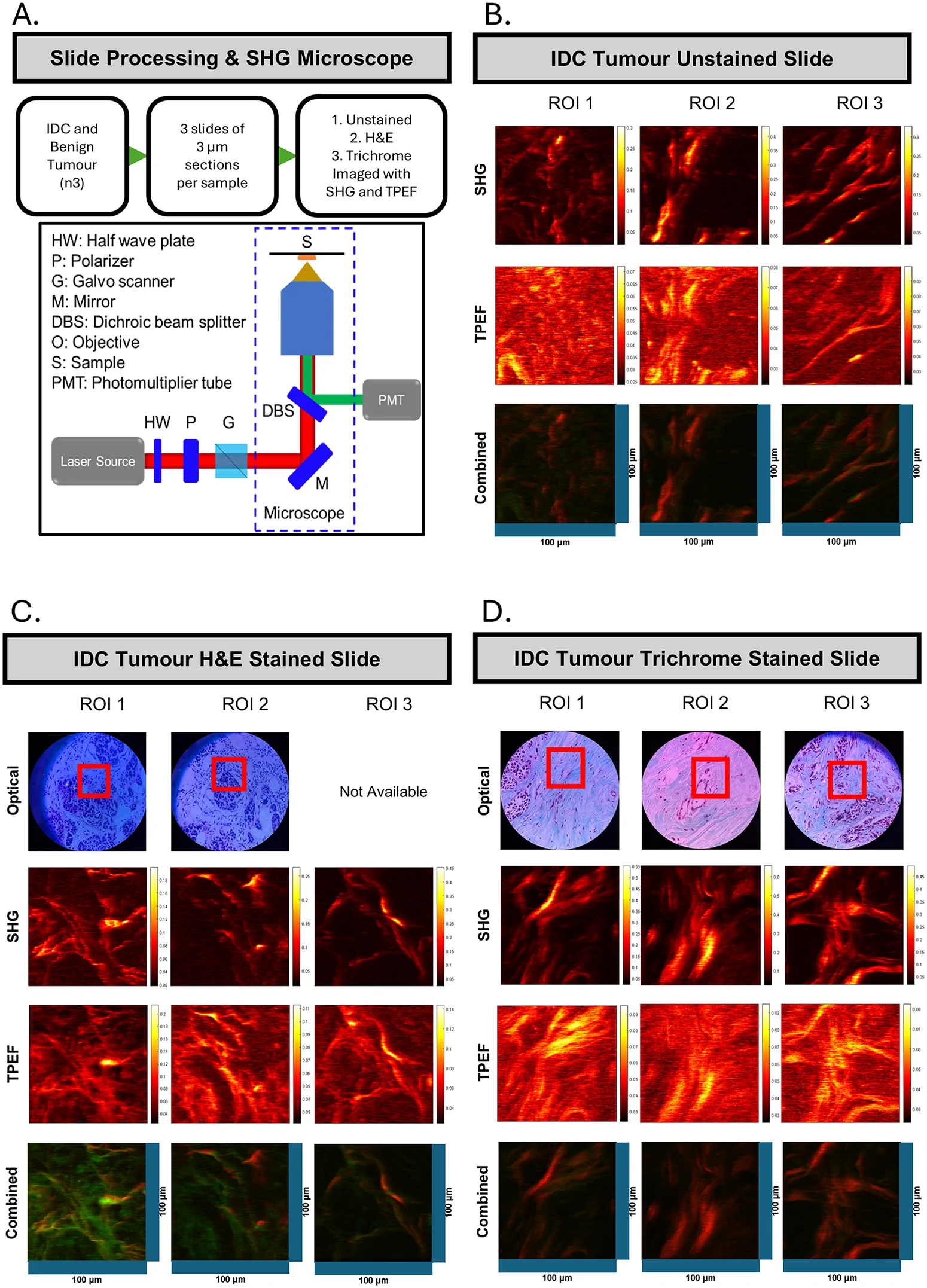
Sample processing and SHG signal detection in three tumour samples. (A) Flow chart describing sample processing and imaging with a schematic of the SHG microscope. Three 100 x 100 µm^2^ ROIs of the IDC High Collagen sample were imaged using SHG microscopy for (B) Unstained Slides, (C) H&E stained and (D) Masson Trichrome stained Slides. Optical references are shown along with SHG and Two-Photon Excitation Fluorescence (TPEF) images acquired for the same ROIs for comparison. No optical references are available for the unstained slides. The red signal is from the SHG channel and green from TPEF in the combined images.

These results confirm that Trichrome staining does not degrade or interfere with the SHG signal, establishing that these routinely stained slides are compatible with SHG microscopy and are suited for cross-modality image analysis.

### SHG images from H&E and Trichrome stained tumour tissue samples

Since unstained slides did not provide any visual reference for confirming accuracy of fibre detection, they were omitted from further imaging and analysis. To visualise macroscopic spatial collagen architecture, we captured larger tissue regions with SHG microscopy. We generated 470 × 470 µm^2^ stitched ROIs across the three samples. Two such ROIs were imaged on H&E and Trichrome-stained slides of benign tumour (Fig 2A), IDC tumour with low collagen (Fig 2B) and IDC tumour with high collagen deposition (Fig 2C). The corresponding SHG image is outlined on the brightfield reference image (Fig 2). While the SHG signal values for both H&E and Trichrome had comparable magnitudes we chose to further analyse Trichrome ROIs since Masson’s Trichrome is collagen specific compared to H&E allowing for comparison of spatial metrics of the collagen fibres.

**Fig 2:**
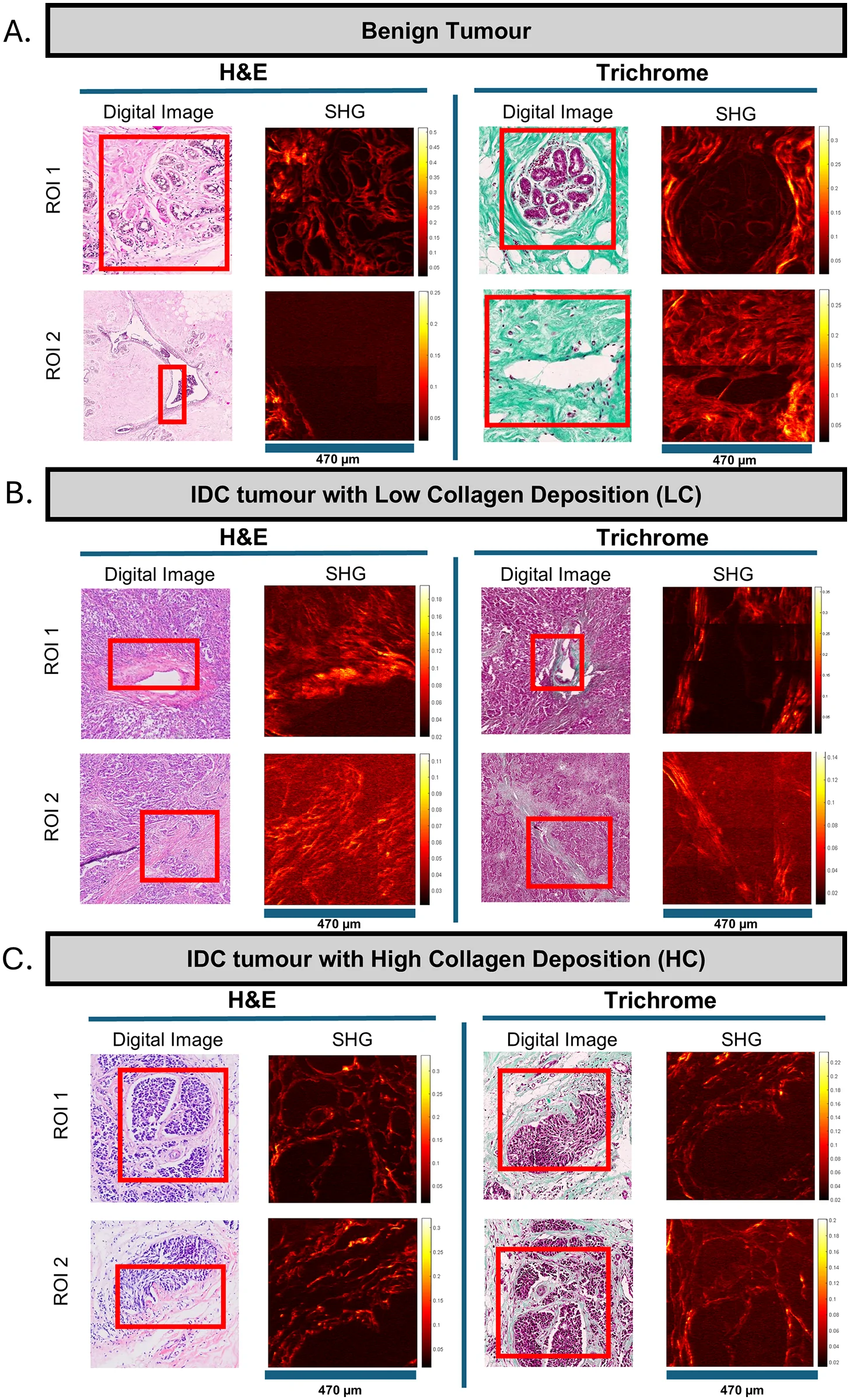
H&E and Trichrome stained slides imaged with SHG microscopy. 25 fields of view (FOVs, a 5 × 5 grid) with dimensions 103 × 103 µm^2^ were imaged with SHG. FOVs were then stitched to generate 470 × 470 µm^2^ ROIs. Such two independent ROIs were imaged from H&E and Trichrome Stained Slides each. The two ROIs imaged with SHG, with their corresponding H&E region is shown in (A) for Benign tumour, (B) for IDC tumour with Low Collagen deposition, (C) IDC tumour with High Collagen deposition. Note that all ROIs are imaged at the same on-sample power of 1.75 mW.

### Collagen fibre visualisation with SHG imaging and digital imaging of Masson Trichrome stain slides

To further compare the two modalities of collagen fibre imaging, SHG and brightfield microscopy and quantify collagen parameters, the ROIs imaged with SHG microscopy were identified and cropped out from the digital whole slide image. To digitally identify Trichrome-stained collagen fibre content, the digital image ROIs were deconvoluted. Two ROIs each of digital image, corresponding deconvoluted binary image and the SHG image are shown for benign tumour (Fig 3A), IDC tissue with low collagen (Fig 3B) and IDC tissue with high collagen (Fig 3C). In regions with dense, bundled collagen, such as the high-collagen IDC ROIs, the SHG signals were strong with minimal noise, matching the digital representation. However, in regions with diffuse or sparse collagen deposition, such as benign ROI 1 and low-collagen ROI 2, the SHG signal suffers data loss. SHG image showed a considerable amount of noise for low-collagen ROI2, likely due to the low amount of collagen deposition among the cellular structures and was excluded from image analysis. This was also analytically observed when assessing alignment of fibres, where noise overpowered the fibre signal detection (Fig 6M). In contrast, the digitally deconvoluted images showed granular details of diffuse and sparse deposition intact.

**Fig 3:**
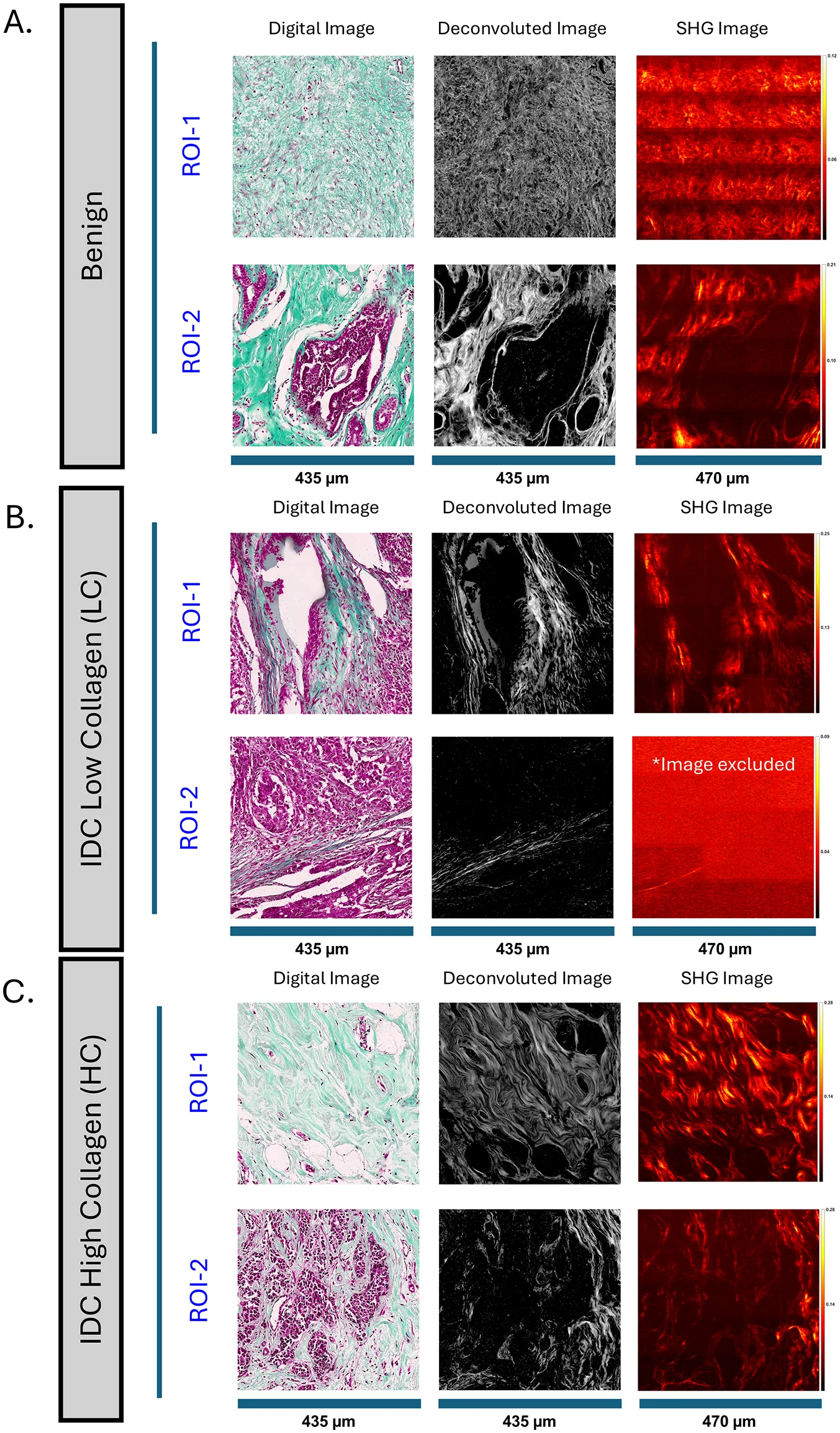
Collagen fibre visualisation with SHG imaging and digital imaging of Masson Trichrome stain slides. The same ROIs that were imaged with SHG microscopy were selected from the digital whole slides scanned image of a Trichrome stained slide and processed with ImageJ for collagen parameters measurements. Corresponding ROIs of 435 × 435 µm^2^ size from the Masson Trichrome images, deconvoluted image of the digital image from the ImageJ pipeline and 470 × 470 µm^2^ stitched image from SHG microscopy are shown in (A) for benign, (B) for IDC tumour with low collagen deposition, (C) IDC tumour with high collagen deposition. Note that all ROIs are imaged with the same on-sample power. *IDC tumour with Low Collagen deposition ROI 2 was excluded from analysis due to poor Signal-to-Noise Ratio (SNR).

This visual comparison highlights a major advantage of the digital pathology approach, as it demonstrates the ability to reliably capture collagen structures in tissue regions where the SHG gold standard could not give a reliable signal.

### Analysis of collagen fibre parameters from SHG microscopy images

We quantified SHG signal intensity and spatial features from 25 FOVs within ROIs of the selected samples to establish a ground-truth baseline of collagen architecture (Fig 4A). We evaluated intra-regional heterogeneity in collagen architecture. Because our objective is methodological validation rather than biological inference, the matched ROIs (not the patients) are the unit of comparison. The 25 FOVs within each ROI are treated as repeated (technical) measurements that characterise that region and are not interpreted as independent biological replicates; accordingly, no patient-level or biological generalisation is drawn from them. All statistical comparisons are therefore made across the matched ROIs, to test whether the analysis pipelines resolve inter- and intra-regional differences in collagen architecture.

**Fig 4:**
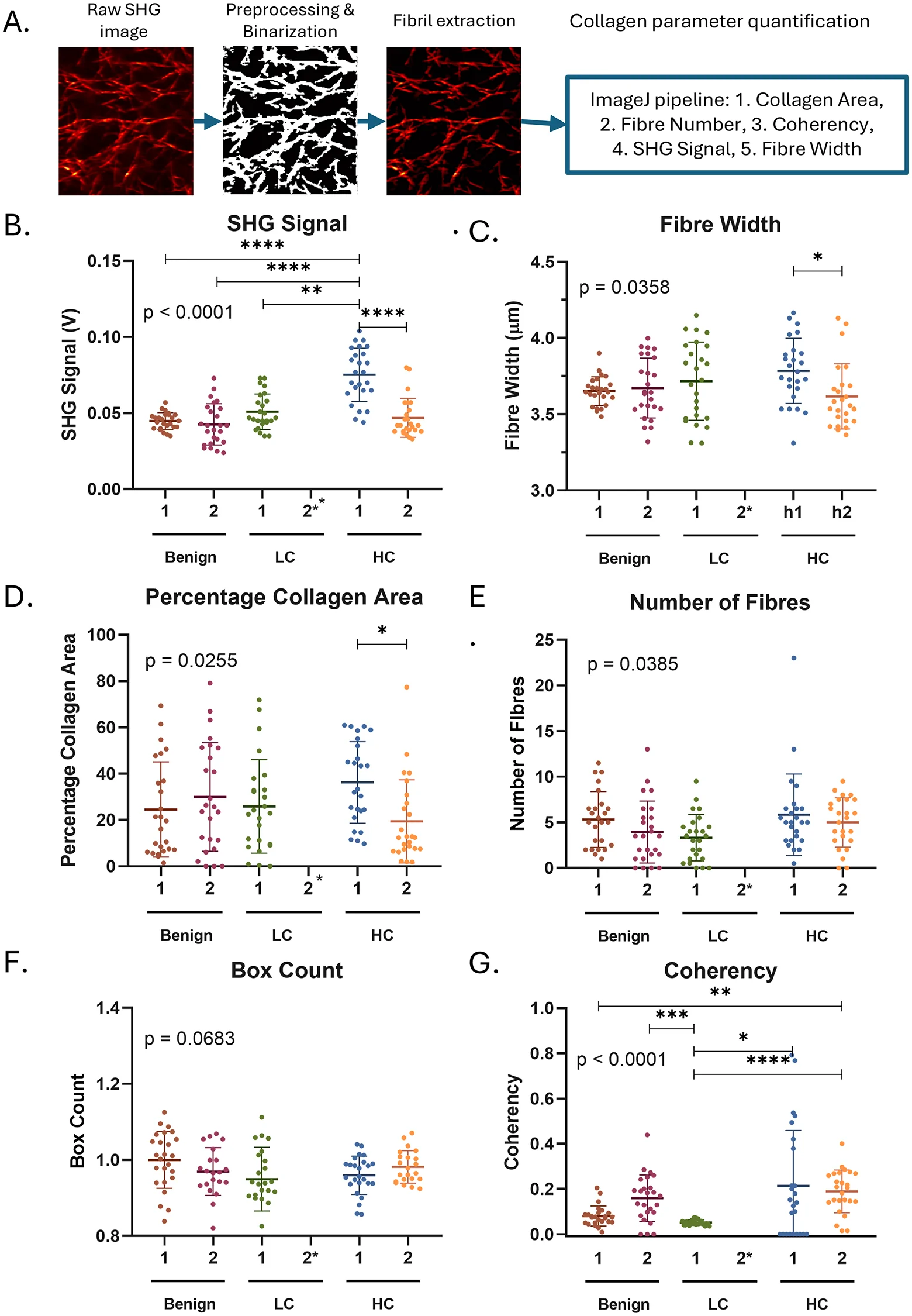
Collagen architectural parameters analysed from SHG images. (A) Analysis workflow computing of collagen deposition parameters from an SHG Images, Cross-ROI comparison of (B) SHG signal, (C) fibre width. Both SHG signal and fibre width were computed using ImageJ. (D) percent collagen area, (E) number of fibres, (F) box count, (G) coherency were computed from SHG images using TWOMBLI macro. Kruskal-Wallis test, along with Dunn’s Multiple Comparison test was performed across all selected ROIs. * ROI 2 of Low Collagen was excluded from analysis.

The average of SHG signal values of the pixels corresponding to the collagen fibre deposition in each FOV was measured. This mean signal differed significantly across selected ROIs (Kruskal-Wallis test, p < 0.0001). High collagen ROI 1, in particular, had a significantly higher mean SHG signal than both benign ROIs (Dunn’s Multiple Comparison test, vs ROI 1 p < 0.0001, vs ROI2 p = 0.0016), low collagen ROI 1 (p < 0.0001) and high collagen ROI 2 (p < 0.0001, Fig 4B). There was also a significant difference in the measurements of fibre width (p = 0.0358, Fig 4C) and percentage collagen area (p = 0.0255, Fig 4D) across ROIs. For both metrics, high collagen ROI 1 and ROI 2 were significantly different, indicating intra-regional heterogeneity (p < 0.05). There were significant differences in the estimated number of fibres across all ROIs (p = 0.0385, Fig 4E). However, this was not the case for box count, measured to evaluate deposition complexity (p = 0.0683, Fig 4F). Our visual observations of SHG data loss were confirmed with ROIs yielding values below 1. Lastly, fibre alignment, quantified with coherency, significantly differed across ROIs (p < 0.0001). Low collagen ROI 1 had significantly less coherency than both benign ROI 1 (p < 0.001) and high collagen ROI 1 (p < 0.0001, Fig 4G).

These observations indicate that the selected ROIs for analysis are highly heterogenous for collagen architecture, with the presence of intra-regional heterogeneity, establishing a favourable setting for our cross-modality comparison.

### Collagen fibre parameter outputs analysed from digital images

In parallel to SHG microscopy, digital image ROIs were analysed using the TWOMBLI pipeline (Fig 5A). Consistent with our SHG analysis, 6 selected ROIs were treated as the statistical unit of analysis to evaluate the pipelines’ sensitivity to intra-regional collagen architecture heterogeneity.

**Fig 5:**
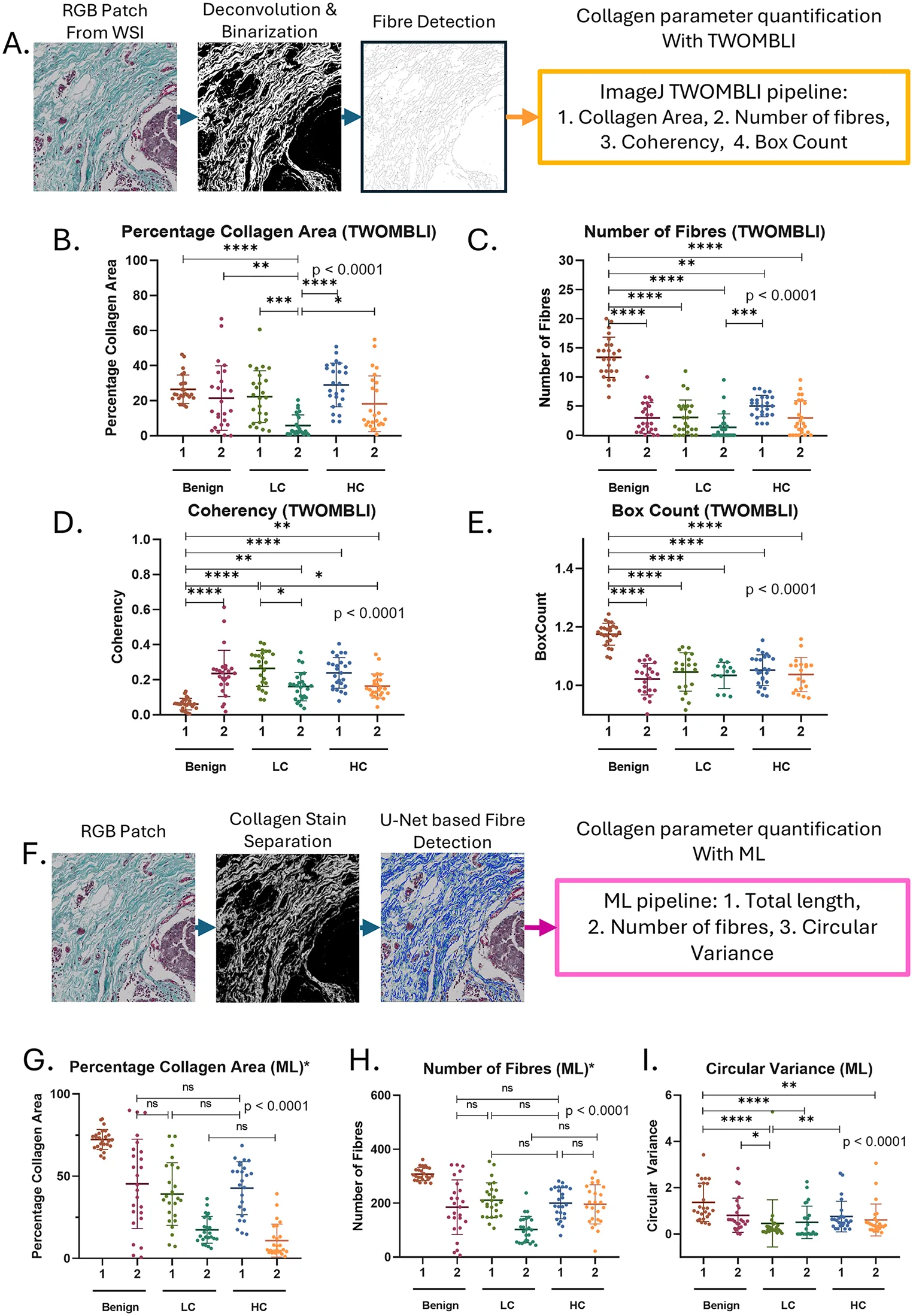
Collagen architectural parameters analysed from digital images. (A) Analysis workflow computing of collagen deposition parameters from a digital image, Cross-ROI comparison of (B) percentage collagen area, (C) number of fibres, (D) coherency, (E) box Count quantified using TWOMBL. (F) Analysis workflow computing of collagen deposition parameters from a digital image using ML. Cross-ROI comparison of (G) percentage collagen area, (H) number of fibres and (I) circular variance quantified using the ML pipeline on the digital image. Kruskal-Wallis, along with Dunn’s Multiple Comparison test was performed across all selected ROIs. *Only non-significant comparisons shown, rest have p-values ranging from <0.01 to <0.0001.

Using the TWOMBLI Image J pipeline, the percentage collagen area differed significantly across the 6 ROIs (Kruskal-Wallis, p < 0.0001, Fig 5B). Similarly, there was significant difference across ROIs for estimated number of fibres (Fig 5C), coherency (Fig 5D), and box count (Fig 5E) with p < 0.0001 for all. The pipeline detected significant differences between benign ROI 1 and benign ROI 2 (p < 0.01) for these four metrics. Notably, benign ROI 1 shows to have a higher number of fibres and box count, with a lower coherency than the other ROIs, in alignment with the visual observation of diffuse deposition.

The digital ROIs were also analysed with a machine learning (ML) pipeline (Fig 5F). This analysis also showed similar consistent and robust trends. The percentage collagen area calculated by this pipeline differed significantly across regions (p < 0.0001, Fig 5G). Significant intra-regional differences were observed between ROI 1 and 2 of the benign sample (p = 0.0042), low collagen sample (p = 0.0261) and high collagen sample (p < 0.0001). The estimated number of fibres from the ML pipeline differed across ROIs overall (Kruskal-Wallis, p < 0.0001), although not all pairwise comparisons reached significance (Fig 5H); this metric showed the weakest concordance between the ML pipeline and SHG (see Fig 6E). Furthermore, for circular variance, the measure for angular fibre disorder, significant architectural differences were seen across the ROIs (p < 0.0001), with the diffuse benign ROI 1 have significantly more circular variance than most ROIs (p < 0.001, Fig 5I).

**Fig 6:**
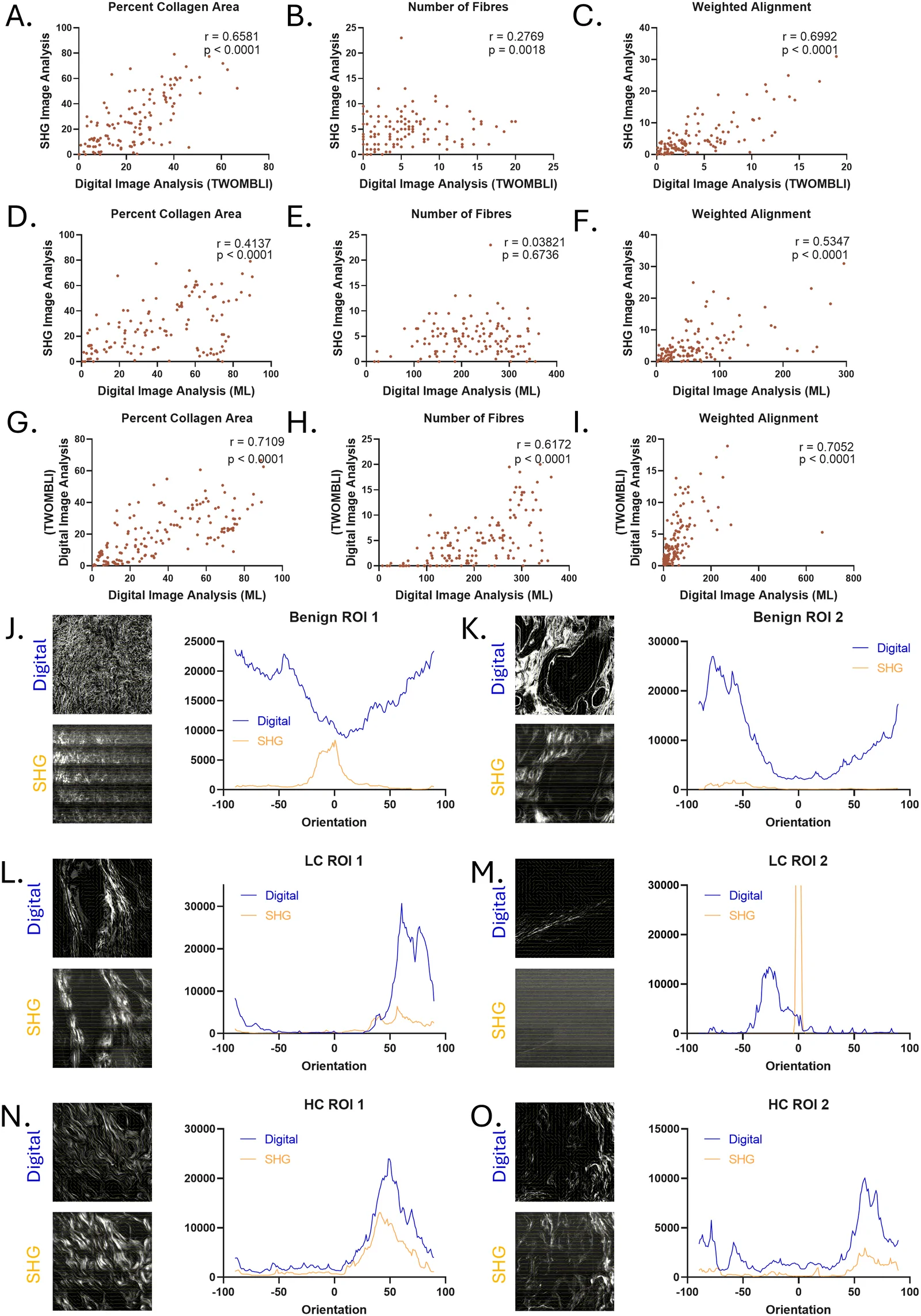
Correlation between collagen deposition parameter measurements from SHG images and digital images. Spearman Correlation of (A) percentage collagen area, (B) number of fibres, (C) weighted alignment measured using TWOMBLI on SHG images and digital Images of Masson Trichrome stained slides. Spearman Correlation of measurements from using TWOMBLI on SHG images and using the ML pipeline on digital images of Trichrome stained slides for (D) collagen area %, (E) number of fibres, (F) weighted alignment Low Collagen ROI 2 is excluded from the correlation analysis. Spearman Correlation of measurements from digital images of the Trichrome stained slide using both the TWOMBLI and ML pipeline for (G) collagen area %, (H) number of fibres, and (I) weighted alignment. Histogram of orientation frequency of collagen deposition with vector fields of the digital and SHG images for (J) Benign ROI 1, (K) Benign ROI 2, (L) Low Collagen ROI 1, (M) Low Collagen ROI 2, (N) High Collagen ROI 1, (O) High Collagen ROI 2. Orientation measurements done on SHG and Digital Images of Masson Trichrome Stained slides.

These findings confirm that utilizing either conventional macros or ML pipelines on accessible digital WSI images, can independently and robustly detect inter- and intra-regional differences in collagen spatial architecture, similar to the SHG gold standard.

### Comparison of collagen deposition quantification with two imaging modalities and three analysis pipelines

Collagen metrics obtained from digital Trichrome images using two image-analysis pipelines were compared with measurements derived from SHG microscopy image analysis using Spearman correlation to determine whether the digital image analysis can reliably approximate the SHG gold standard. To assess potential exclusion bias, correlation analyses were performed both by including all 6 ROIs (150 FOVs) and by excluding low collagen (LC) ROI 2 (125 FOVs), which previously showed SHG data loss.

Between SHG and digital image analysis pipeline using TWOMBLI (Fig 6A-C), there was a significant and strong positive correlation for collagen area percentage (r = 0.6581 and p < 0.001, Fig 6A) and weighted alignment (r = 0.6992, p < 0.0001; Fig 6C), alongside a significant but weak positive correlation for number of fibres (r = 0.2769, p = 0.0018; Fig 6B). Crucially, when LC ROI 2 was included, these strong correlations were preserved across all metrics (Area: r = 0.6995, p < 0.001; Alignment: r = 0.6683, p < 0.001; Fibres: r = 0.2736, p = 0.0007, S1 Data, Sheet 3).

Similarly, the ML pipeline showed robust cross-modality concordance. Excluding LC ROI 2, there was a significant and positive correlation for collagen area percentage (r = 0.4137, p < 0.0001; Fig 6D) Weighted alignment of fibres (calculated as percentage collagen area divided by the circular variance, representing degree of disorder) was also positively correlated with the weighted alignment calculated from the SHG image (r = 0.5347, p < 0.0001; Fig 6F). However, number fibres did not show concordance. Like the previous set of comparisons, correlations were still strong and significant on including LC ROI 2 (Area: r = 0.5717, p < 0.0001; Alignment: r = 0.5225, p < 0.0001; Fibres: r = 0.2119, p = 0.0095, S1 Data). Furthermore, when the two independent digital image analysis pipelines were compared with each other, they exhibited strong positive and highly significant correlations across all parameters, including area (r = 0.7109, p < 0.0001, Fig 6G), number of fibres (r = 0.6172, p < 0.0001, Fig 6H), and weighted alignment (r = 0.7052, p < 0.0001, Fig 6I).

Histograms of orientation frequencies show a more granular perspective of how orientation measured on Trichrome stained slides differs for each ROI across imaging modalities. A concentration of frequencies was distributed around 0° from the SHG image of benign ROI 1, this distribution differs from that of the digital image of the same ROI (Fig 6J). This indicates more noise is being picked up for this ROI, likely due to data loss due to the diffuse nature of collagen deposition. The distribution of the SHG image orientation values slightly aligns with that of the digital image for benign ROI 2, although the magnitude of the digital image orientation is much higher (Fig 6K). The distributions for the low collagen ROI 1 align, with differing magnitudes (Fig 6L). There is a high amount of noise in the SHG image of low collagen ROI 2 as seen by the peak at 0°, whereas the distribution peaks at -25° for the digital image (Fig 6M). The higher noise could be due to the overall low amount of collagen in this ROI. The distributions of orientations from SHG images align strongly with those of the digital images for both ROIs of high collagen (Fig 6N and O).

Together, these correlation analyses validate that accessible digital WSI pipelines can accurately and robustly quantify the spatial features of collagen deposition architecture performing in concordance with SHG microscopy, while also being able to successfully capture regions with scanty or diffuse collagen deposition.

Overall, the two imaging modalities: SHG microscopy and brightfield digital microscopy images and their analysis pipelines were compared for the data output with potential relevance to clinical diagnosis (Table 1). While SHG microscopy has the advantage of directly visualising fibrillar collagen on both unstained and stained tissue sections, the technique necessitates specialised instrumentation and experienced personnel for operation. On the other hand, studying collagen using brightfield microscopy relies on stains that are routinely used by pathologists to visualise clinical fibrosis which involves collagen deposition. The SHG image analysis pipeline demonstrated a higher accuracy of collagen quantification for bundled and aligned collagen fibres, specifically measuring molecular ordering the SHG signal metric. While the brightfield digital image analysis effectively detected broader heterogeneity and was not limited to specific architecture of collagen fibres, quantifying wider range of collagen architectural parameters. Thus, the brightfield images analysed with digital pathology tools may be able to prognosticate a broader range of architecture presenting at the clinic. At a diagnostic setting, a brightfield microscope is routinely available, unlike an SHG microscope. In addition, our digital pathology analysis pipeline can be made accessible to path lab computers quite easily in today’s digital age, as it is designed to operate entirely on Central Processing Units (CPUs) without requiring high-end Graphics Processing Unit (GPU) acceleration. It avoids loading the entire WSI into memory, and relies on tile-based multiprocessing across multiple CPU threads. This approach efficiently manages resources and runs on moderate hardware (e.g., 8 CPU threads and 32 GB RAM). Similarly, the TWOMBLI analysis can also be performed on machines with the same hardware. Ultimately, brightfield analysis of Trichrome-stained slides can leverage the scalability and accessibility of established frameworks for clinical investigations.

**Table 1:** Comparison of two modalities of collagen fibre imaging and measurements.

	SHG Imaging	Brightfield imaging with digital pathology
**Tissue processing and Staining requirements**	Works on both, stained as well as unstained tissue sections	Routine staining protocol is followed
**Imaging Microscope**	Specialized microscope requiring experienced personnel to operate	Simple Brightfield microscope
**Collagen Parameter Quantification**	a. Higher accuracy with bundled and aligned fibres, not otherwise. b. Three parameters of collagen architecture are measured: signal intensity, fibre width and collagen area.	c. Detects range of heterogeneity in collagen deposition. d. Can compute complex architecture of collagen such as cross-linking and fibre alignment, along with the area, fibre number.
**Clinical Diagnostic Utility**	None. SHG imaging is used for research purposes at the moment.	Trichrome stain in routinely used for diagnostics of fibrotic diseases.

## Discussion

Collagen organization within the tumour microenvironment plays an important role in tumour progression, invasion, and patient prognosis. SHG microscopy has emerged as a powerful technique for visualizing and quantifying fibrillar collagen architecture due to its ability to selectively detect non-centrosymmetric collagen fibres without exogenous labelling. However, the high cost, specialized instrumentation, and limited scalability at a diagnostic setting of SHG microscopy restrict its widespread implementation in routine pathology workflows. In this study, we systematically evaluated whether quantitative collagen features derived from digitally scanned Masson–Goldner’s Trichrome–stained histopathological sections can approximate measurements obtained from SHG microscopy, which is considered as a gold standard for collagen architectural measurements.

We first assessed the compatibility of SHG imaging with Trichrome-stained sections by comparing collagen fibre detection across unstained, H&E-stained, and Trichrome-stained tissue sections. Studies exploring SHG imaging of H&E stained slides have shown that H&E staining is compatible with SHG microscopy [8,18], however it is also reported that the dyes can decrease SHG signal intensity [19] indicating stain interference. A key observation from this study is that SHG imaging remains compatible with Trichrome-stained tissue sections. While SHG signals were observed across unstained, H&E-stained, and Trichrome-stained sections, and yielded similar collagen features. This observation indicates that Trichrome staining does not interfere with collagen SHG detection and in fact may enhance contrast due to improved optical definition of collagen bundles. This compatibility enabled direct comparison of two modalities, namely: SHG imaging with digital brightfield image analysis using the same tissue sections and matched regions of interest with visual reference.

Following this validation, matched regions of interest from the same tissue sections were analysed using whole-slide digital imaging and two independent computational pipelines, an ImageJ-based workflow and a machine learning–based analytical framework. There have been highly impactful advances in the field of digital pathology following the development of sophisticated foundational models like CHIEF and PROGPath [20,21]. While these models demonstrate exceptional performance in extracting patch-level semantics and disease patterns for prognostic tasks, the quantitative assessment of extracellular matrix architecture presents a distinct computational challenge. Extracting spatial metrics from collagen fibres requires a focus on pixel-level geometry and sensitivity to anisotropic structures. This necessitates our domain adapted approach utilizing a U-Net model developed by Park and colleagues, 2023 [17].

We examined the extent to which collagen features derived from routine histopathological imaging can approximate those obtained through SHG microscopy and evaluate the potential of digital pathology–based approaches as more accessible alternatives for quantitative collagen analysis in tumour tissues. Through careful alignment of tissue features, we extracted ROIs from the WSIs of the Trichrome stained slides corresponding to the ROIs imaged using SHG from the same slide, making an ROI-to-ROI comparison possible. We observed that although the SHG signal output was satisfactory in the presence of bundled collagen fibres, the deconvoluted digital image of all samples was a better representative of the collagen fibres seen in the digital image than the SHG image. This may be because pixel size of SHG imaging (0.44 µm per pixel) is more than the digital image and the deconvoluted image (0.253 µm per pixel) along with the reduced noise from deconvolution of the digital image. It is worthy to note the high resolution of the digital scanner obtained a matter of minutes, indicating an improvement in brightfield microscopy technology of histological tissue. We deliberately did not resample the two modalities to a common pixel size: down-sampling the higher-resolution digital image to match the SHG resolution would blur fine fibres and lose information. Because the metrics compared here are extracted across fibres that span many pixels, they are largely insensitive to pixel size, enabling valid comparison across modalities of differing resolution and field of view.

Quantitative analysis demonstrated that collagen structural parameters derived from digital Trichrome images show strong concordance with SHG-derived measurements. Metrics describing collagen abundance and spatial organization, including collagen deposition area, fibre number, and fibre alignment, showed statistically significant correlations across imaging modalities. In particular, collagen area and fibre alignment exhibited strong positive correlations between SHG analysis and digital image analysis using the TWOMBLI pipeline [13]. These findings indicate that digital histopathological images can capture spatial characteristics of collagen architecture that are comparable to those obtained using SHG microscopy. Furthermore, the quantifications from the digital image captured the differential presentation of collagen deposition across ROIs while SHG images showed loss of data at lower ranges of collagen deposition.

Two independent digital image analysis approaches: an ImageJ-based TWOMBLI workflow and a machine learning–based computational pipeline, produced broadly consistent quantitative outputs. Strong correlations between these pipelines for collagen area, fibre number, and alignment-related metrics demonstrate the robustness and reproducibility of collagen quantification from digital histopathology images. The machine learning–based framework further enabled automated segmentation of collagen fibres and quantification of fibre orientation disorder using circular variance, illustrating the potential for scalable and automated collagen analysis in whole slide images.

These findings might have important implications in the context of breast cancer stromal biology. Increasing evidence indicates that the extracellular matrix, particularly fibrillar collagen organization, actively regulates tumour behaviour by influencing tumour cell migration, mechanotransduction, and immune cell infiltration. Aligned collagen fibres surrounding tumours have been associated with invasive tumour fronts and poor clinical outcomes [6,7]. Quantitative analysis of collagen architecture therefore represents an important avenue for identifying stromal biomarkers of tumour progression. By demonstrating that spatial collagen metrics extracted from routine histopathological images correlate with SHG-derived measurements, our findings suggest that potentially clinically relevant features of tumour collagen organization may be captured using widely available digital pathology infrastructure.

A major advantage of the digital pathology–based approach lies in its greater scalability and compatibility with routine clinical workflows. The larger field-of-view available through whole slide imaging also enables evaluation of stromal heterogeneity across tumour sections, which is difficult to capture using SHG microscopy alone. As digital pathology platforms become increasingly integrated into diagnostic practice, computational analysis of collagen architecture from routinely stained slides could enable large-scale, standardized evaluation of stromal features across clinical cohorts.

The present study has several limitations. First, the analysis was performed on a limited number of tissue samples. Validation in larger patient cohorts will be necessary to confirm the robustness and generalisability of the observed correlations to support clinical relevance. Second, differences in spatial resolution between SHG microscopy and brightfield digital images required approximate matching of regions of interest rather than exact pixel-level correspondence. Due to the lack of paired SHG and Trichrome images, we were unable to perform fibre‑level validation of the domain‑adapted method. Our validation was limited to statistical concordance with TWOMBLI‑derived metrics, which supports the proof‑of‑concept but does not establish direct fibre‑level accuracy. Additionally, while digital image analysis captured several spatial features of collagen organization, it cannot directly measure nonlinear optical properties of collagen fibres, such as SHG signal intensity, that reflect molecular ordering within collagen bundles. Also, estimating spatial parameters of collagen fibres from 2D tissue sections inherently introduces sectioning artefacts. In this study all ROI and analysis pipelines are affected by this equally. However, our approach of imaging larger tissue areas helps minimise this effect. Finally, validation across multi-institutional cohorts would be necessary to confirm robustness of these tools against diverse real-world staining practices.

Despite these limitations, this study provides an important methodological advance by demonstrating that collagen architectural features derived from routine histopathological images can approximate SHG-derived collagen measurements. To our knowledge, this work represents one of the few systematic comparisons integrating nonlinear optical imaging, conventional image analysis, and machine learning–based computational pipelines for quantitative collagen analysis within matched tissue regions.

In conclusion, our findings demonstrate a proof-of-concept that collagen structural features quantified from digitally scanned Masson–Goldner’s Trichrome-stained sections show strong concordance with SHG-derived measurements of collagen organization. Computational analysis of routine histopathology images using both conventional image processing and machine learning approaches could enable scalable quantification of collagen architecture while maintaining consistency with SHG-based measurements. As stromal features increasingly emerge as important biomarkers in tumour biology, digital pathology–based collagen quantification may provide a accessible strategy for integrating extracellular matrix analysis into routine cancer diagnostics and translational research.

## Supporting information

S1 Fig Representative annotation on whole slide images (WSI). Thumbnail images of whole slide images (WSIs) uploaded to PathPresenter with annotations marking regions of interest on H&E and Trichrome Stained slides of (A) Benign Tumour, (B) IDC tumour with Low Collagen deposition based on Masson Trichrome stain and (C) IDC tumour with High Collagen deposition, for SHG imaging. Scale bars are 7 mm.
(PDF)

S2 Fig USAF calibration of SHG microscope for pixel size, and dimensions of stitched SHG ROI. US Airforce (USAF) Calibration of SHG Microscope Imaging A) Group 5, Element 1, B) Group 5, Element 3, C) Group 5, Element 4, D) Group 5, Element 6. E) Dimensions of imaged FOV, overlap and ROI of Stitched SHG ROI.
(PDF)

S1 Data Calibration, quantification and correlation metrics of collagen fibre parameters. This file contains: 1. USAF Calibration metrics for pixel size calculation of SHG images, 2. quantified measurements of collagen fibre parameters of each patch of the selected SHG and digital ROIs, 3. Spearman correlation metrics of cross modality comparisons with LC ROI2 included and excluded.
(XLSX)
