# Supplementary material for: Quantitative assessment of collagen architecture from routine histopathological images shows concordance with Second Harmonic Generation microscopy
Source: PLOS Digit Health. 2026 Oct 26;5(10):e0001364. doi: 10.1371/journal.pdig.0001364 (PMC13704883; doi:10.1371/journal.pdig.0001364)
Supplement: Supplementary file 1 [file pdig.0001364.s001.pdf]

A.

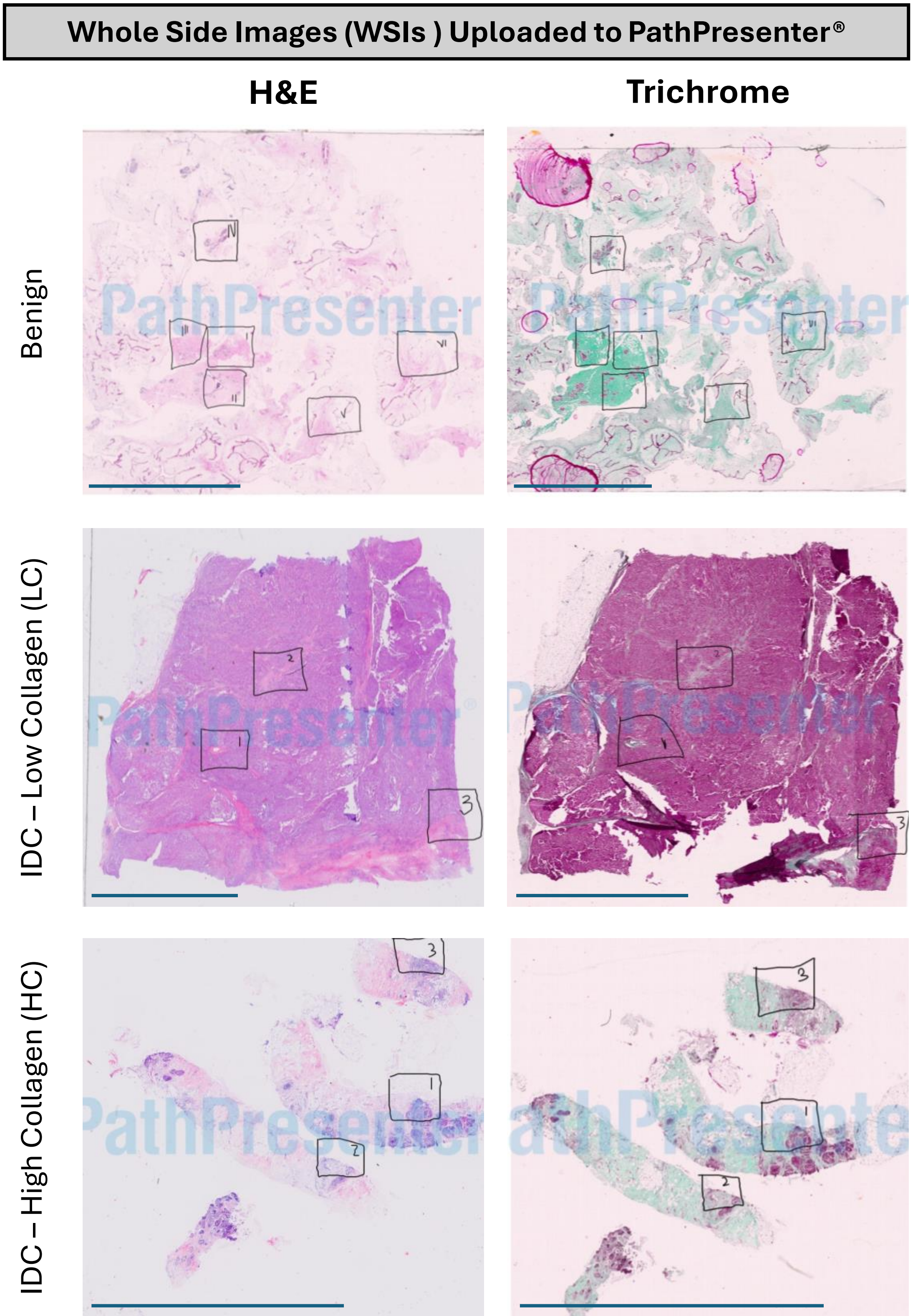

Whole Side Images (WSIs) Uploaded to PathPresenter®
H&E
Trichrome
Benign
IDC – Low Collagen (LC)
IDC – High Collagen (HC)
