# Supplementary material for: Quantitative assessment of collagen architecture from routine histopathological images shows concordance with Second Harmonic Generation microscopy
Source: PLOS Digit Health. 2026 Oct 26;5(10):e0001364. doi: 10.1371/journal.pdig.0001364 (PMC13704883; doi:10.1371/journal.pdig.0001364)
Supplement: Supplementary file 2 [file pdig.0001364.s002.pdf]

A. Group – 5, Element – 1

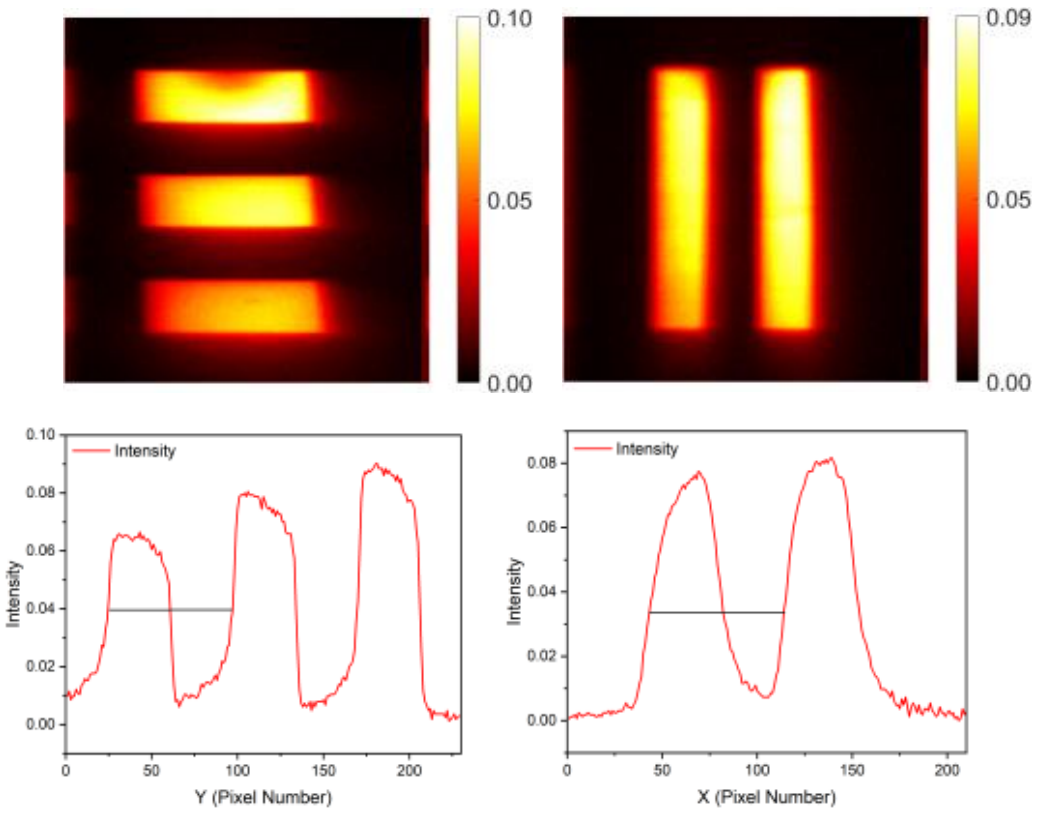

Resolution (lp/mm) = 32
Line pair width (μm) = 1000/32 = 31.25
Pixel size (Y) = 31.25/73 = 0.4281
Pixel size (X) = 31.25/72 = 0.4340
Scan length (X) = 0.4340 × 250 = 108.5 μm
Scan length (Y) = 0.4281 × 250 = 107 μm

B. Group – 5, Element – 3

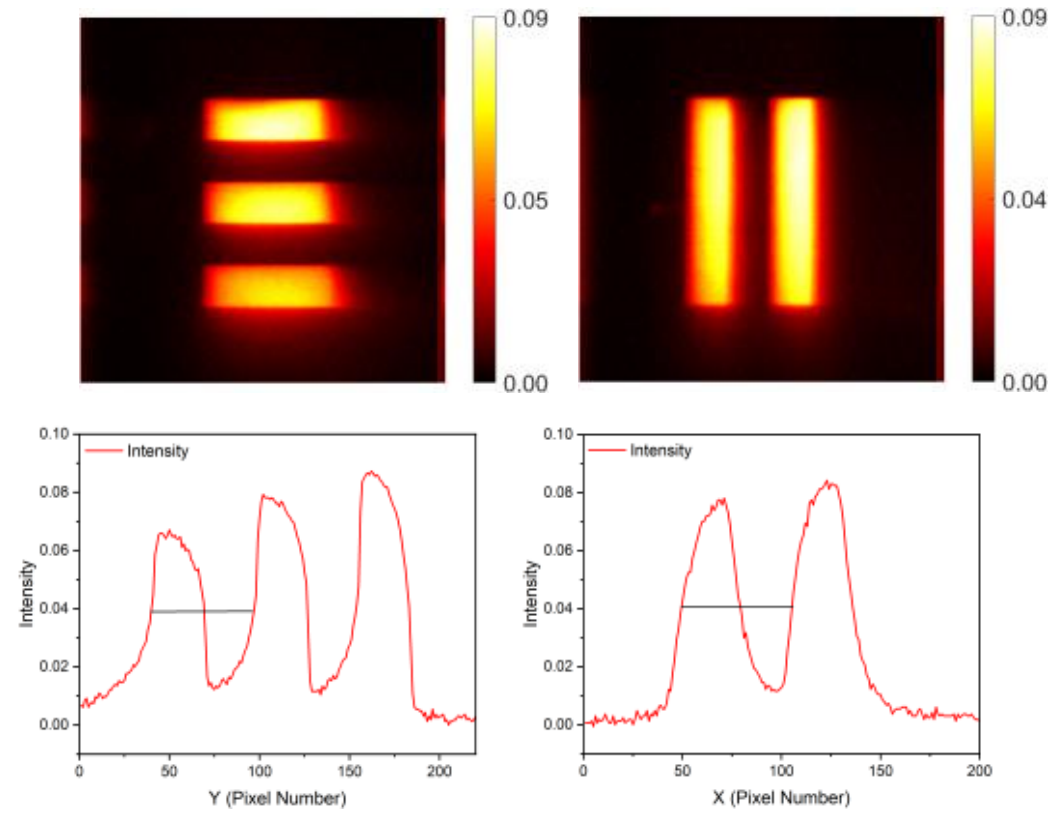

Resolution (lp/mm) = 40.3
Line pair width (μm) = 1000/40.3 = 24.814
Pixel size (Y) = 24.814/57 = 0.4353
Pixel size (X) = 24.814/56 = 0.4431
Scan length (X) = 0.4431 × 250 = 110.7 μm
Scan length (Y) = 0.4353 × 250 = 108.8 μm

C. Group – 5, Element – 4

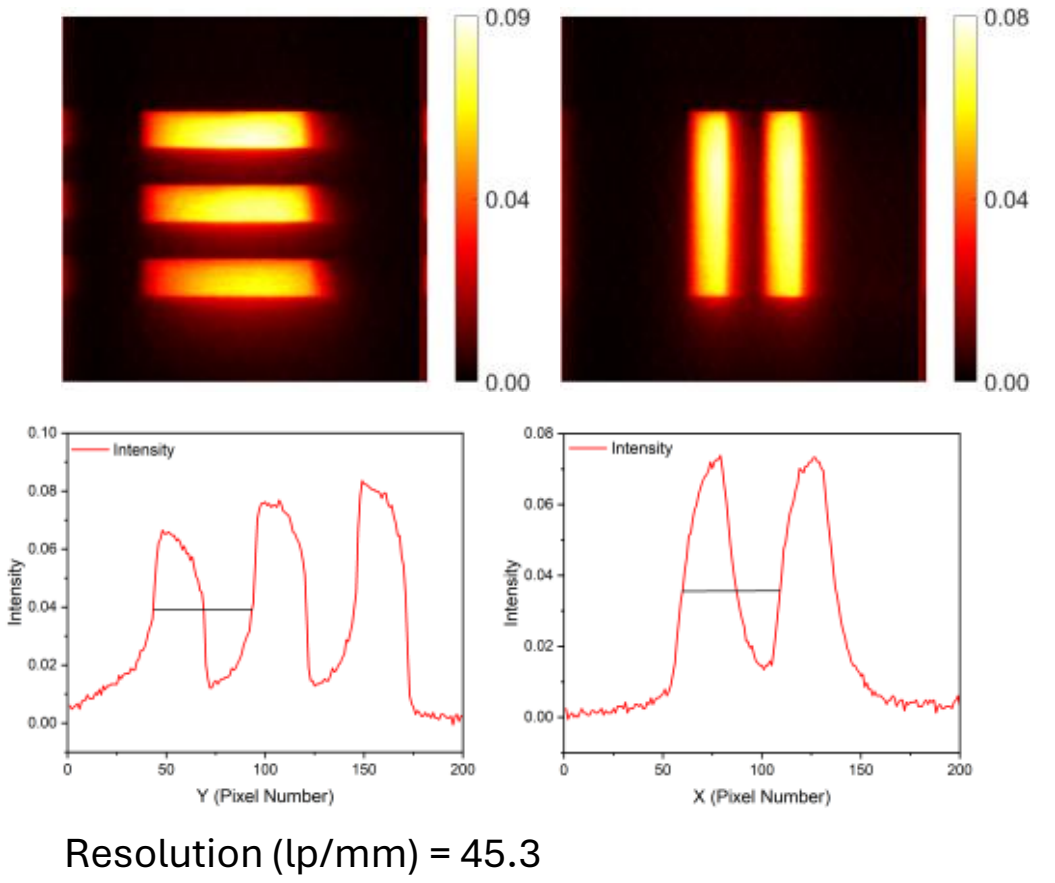

Resolution (lp/mm) = 45.3
Line pair width (μm) = 1000/45.3 = 22.075
Pixel size (Y) = 22.075/50 = 0.4415
Pixel size (X) = 22.075/49 = 0.4505
Scan length (X) = 0.4505 × 250 = 112.6 μm
Scan length (Y) = 0.4415 × 250 = 110.4 μm

D. Group – 5, Element – 6

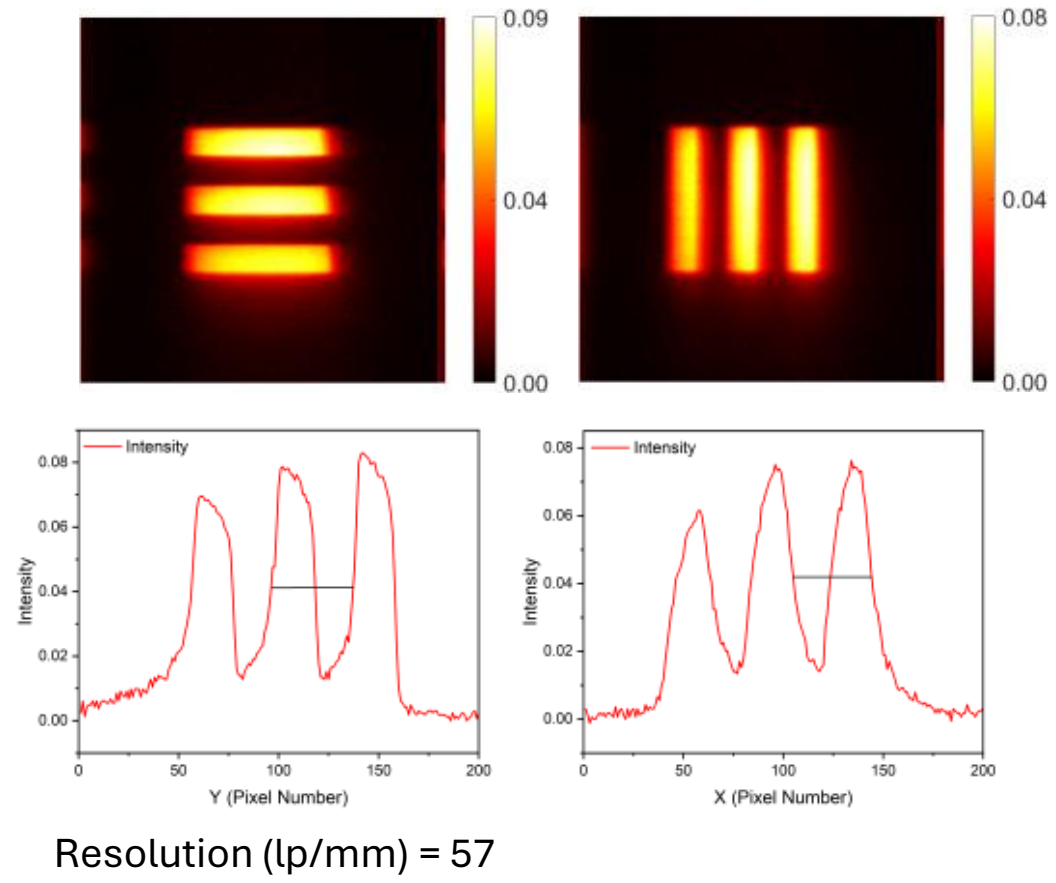

Resolution (lp/mm) = 57
Line pair width (um) = 1000/57 = 17.5439
Pixel size (Y) = 17.5439 /40 = 0.4386
Pixel size (X) = 17.5439 /39 = 0.4498
Scan length (X) = 0.4498 × 250 = 112.4 um
Scan length (Y) = 0.4386 × 250 = 109.7 um

E. Stitched SHG ROI

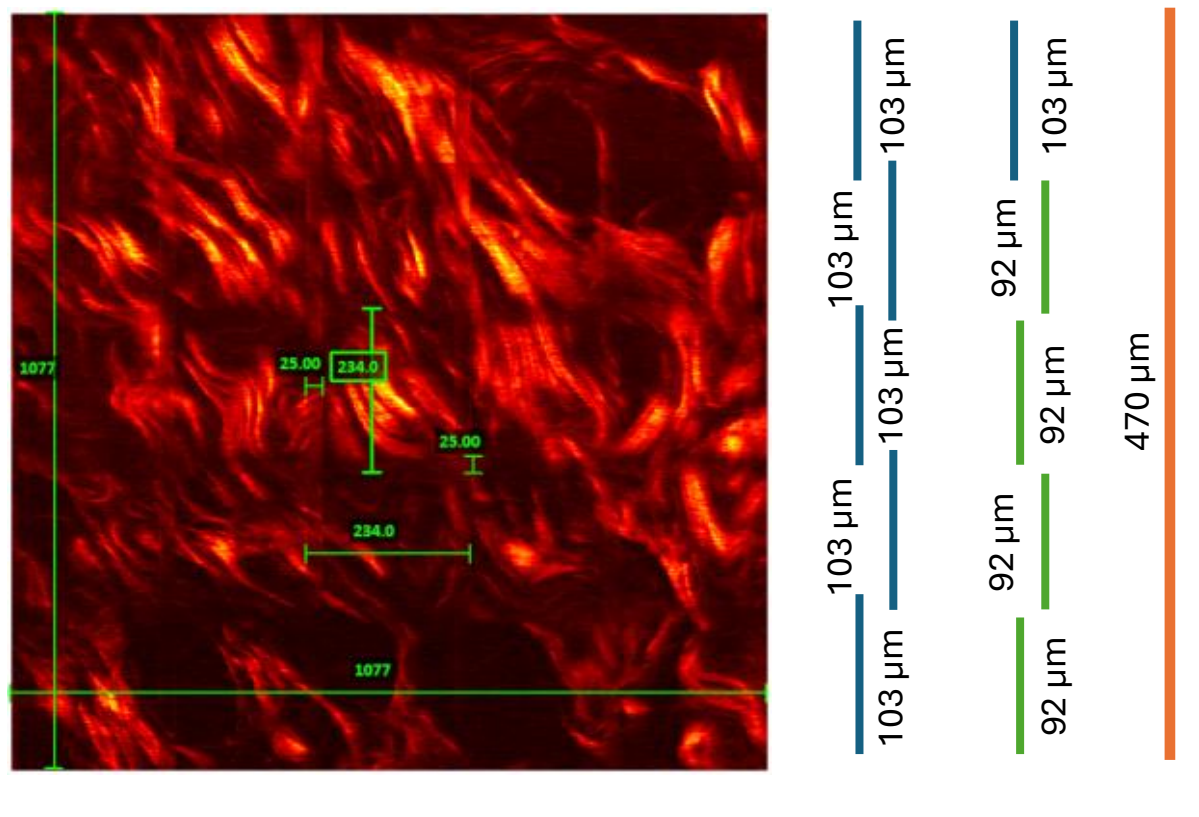

Average Pixel Size from USAF Calibration = 0.4401 ± 0.008 μm

Dimension of	pixels	μm
FOV	234	103
Overlap	25	11
ROI	1077	470
