# Supplementary material for: Quantitative assessment of collagen architecture from routine histopathological images shows concordance with Second Harmonic Generation microscopy
Source: PLOS Digit Health. 2026 Oct 26;5(10):e0001364. doi: 10.1371/journal.pdig.0001364 (PMC13704883; doi:10.1371/journal.pdig.0001364)
Supplement: Supplementary file 3 [file pdig.0001364.s005.pdf]

Response to Reviewers

Manuscript PDIG-D-26-00512

Quantitative assessment of collagen architecture from routine histopathological images shows concordance with Second Harmonic Generation microscopy

Dear Editor and Reviewers,

We thank the Academic Editor and the three reviewers for their careful and constructive evaluation of our manuscript. In preparing this revision we have undertaken substantive additional analysis and rewriting rather than superficial edits. In brief, we have: (i) reanalysed all cross-modality comparisons at the level of matched regions of interest (ROIs) to address concerns about the unit of analysis and pseudo replication; (ii) performed an additional sensitivity analysis in which the previously excluded low-collagen ROI was reintroduced, confirming that the reported correlations are preserved; (iii) substantially expanded the description of the machine-learning pipeline to enable reproducibility, including the domain-adaptation strategy and the use of a previously published, validated pre-trained U-Net model; (iv) reframed the study throughout as a methodological proof-of-concept, tempering claims of clinical readiness and scalability; v) reverified microscope calibration information, created a supplementary figure on the same and corrected dimensions; and vi) rewritten results to follow a strong narrative, improving the readability of the text.

A detailed, point-by-point response to every comment is provided below. Our responses are blue font, following reviewer's comments. All corresponding changes are marked in the accompanying tracked-changes manuscript. References to edits are drawn here in the rebuttal. Page and line numbers cited below refer to the clean (accepted) revised manuscript.

Response to the Academic Editor Comments:

Editor's comments:

1. We note that you received funding from a commercial source: [Bajaj Pvt Ltd] Please provide an amended Competing Interests Statement that explicitly states this commercial funder, along with any other relevant declarations relating to employment, consultancy, patents, products in development, marketed products, etc.

Within this Competing Interests Statement, please confirm that this does not alter your adherence to all PLOS policies on sharing data and materials by including the following statement: ""This does not alter our adherence to PLOS policies on sharing data and materials." (as detailed online in our guide for authors http://journals.plos.org/plosone/s/competing-interests). If there are restrictions on sharing of data and/or materials, please state these. Please note that we cannot proceed with consideration of your article until this information has been declared.

Please update your Competing Interests Statement within the submission form upon resubmission of your manuscript. If you are unable to do so, please email the journal office, and we may make these updates on your behalf.

Response: We would like to bring to notice that we have received philanthropic funds from Bajaj Auto, as corporate social responsibility (CSR) funds, to the "Center for Translational Cancer Research" at Prashanti Cancer Care Mission, Pune. Hence, Bajaj Auto, has no vested or competing interest in the research that is presented here.

2. Some material included in your submission may be copyrighted. According to PLOS's copyright policy, authors who use figures or other material (e.g., graphics, clipart, maps) from another author or copyright holder must demonstrate or obtain permission to publish this material under the Creative Commons Attribution 4.0 International (CC BY 4.0) License used by PLOS journals. Please closely review the details of PLOS's copyright requirements here: PLOS Licenses and Copyright. If you need to request permissions from a copyright holder, you may use PLOS's Copyright Content Permission form.

Please respond directly to this email or email the journal office and provide any known details concerning your material's license terms and permissions required for reuse, even if you have not yet obtained copyright permissions or are unsure of your material's copyright compatibility.

Potential Copyright Issues:

a. Figures 1, 2, 3, 4, 5, 6, 7, S1 Fig: Please confirm whether you drew the images / clip-art within the figure panels by hand. If you did not draw the images, please provide (a) a link to the source of the images or icons and their license / terms of use; or (b) written permission from the copyright holder to publish the images or icons under our CC-BY 4.0 license. Alternatively, you may replace the images with open source alternatives. See these open source resources you may use to replace images / clip-art:

   - https://commons.wikimedia.org
   - https://openclipart.org/

Response: All the images are original, generated by our team. No data or images have been sourced from outside.

b. We do not publish any copyright or trademark symbols that usually accompany proprietary names, eg (R), (C), or TM (e.g. next to drug or reagent names). Please remove all instances of trademark/copyright symbols throughout the text, including PathPresenter®.

Response: We have removed the trademark and copyright symbols from the manuscript, as per the request.

3. We ask that a manuscript source file is provided at Revision. Please upload your manuscript file as a .doc, .docx, .rtf or .tex.

Response: Yes, we have uploaded .doc manuscript source file as a clean version of the revised manuscript.

Editor's other comments: A major revision is reasonable. The authors are expected to complete the revision with additional experiments, discussions and justifications to support their responses to the reviewers. Note that simply using GPT or similar to respond to the challenges literally is not acceptable. Reproducibility is needed.

Response: We appreciate the editor's guidance. The revision is built on genuine additional analysis and manuscript rewriting, as summarised above: ROI-level reanalysis with updated Figures 4 and 5, a new exclusion-bias sensitivity analysis, a substantially expanded and reproducible Methods description of the machine-learning pipeline, corrected imaging resolutions and ROI dimensions verified against the raw acquisition files (submitted as an additional supplementary figure, S2) and a consistent reframing of the

work as a proof-of-concept. We have also expanded the discussion of prior computational-pathology work and improved data and code availability (see Reviewer 2, Comment 7).

Response to Reviewer #1

Summary:

This study explores whether collagen structural features derived from routine histopathological images can approximate those obtained from SHG microscopy, which addresses a problem in digital pathology. However, the novelty is moderate, since similar approaches for collagen quantification from histology have already been reported. The main contribution of this work is the cross-modality validation, rather than proposing a new methodology. The study is generally well designed, especially in terms of multi-pipeline comparison and ROI matching. Nevertheless, it is limited by a very small sample size and the lack of external validation. In addition, the machine learning part needs more detailed description and more comprehensive evaluation. This work is promising, but stronger validation is still needed to support its clinical applicability.

We thank Reviewer #1 for recognising the cross-modality design and multi-pipeline comparison, and for the constructive suggestions that have strengthened the manuscript.

Major concerns

Comment 1. The machine learning pipeline using U-Net combined with clustering is not described in sufficient detail. Several important aspects are missing, including the size of the training dataset, the definition and source of ground truth annotations, the validation strategy, and the performance metrics used such as Dice coefficient and IoU. Without this information, it is difficult to evaluate the reliability and robustness of the model. In addition, the current description does not allow the study to be reproduced. A more complete and transparent description of the machine learning methodology is necessary, and providing the code on GitHub would improve reproducibility.

Response: We agree that the original description lacked the technical depth required for reproducibility, and we have extensively rewritten and expanded the "Digital Image Analysis with ML" subsection of the Methods (revised manuscript, Methods, 'Digital Image Analysis with ML'; page no.: 13–14, line no.: 245–278.) To clarify, our study did not train a new U-Net model from scratch; for this reason, training-specific metrics such as dataset size, ground-truth annotations, validation splits, and Dice/IoU coefficients were not generated here. Instead, we implemented a domain-adaptation approach in which a robust, previously published pre-trained U-Net developed and validated for SHG imaging by Park and colleagues (Park et al., 2023) was employed; the original training and validation metrics are reported in Park et al 2023.

The novelty of our pipeline lies in transforming brightfield Masson–Goldner's Trichrome images into 'SHG-like' inputs that can be segmented by this pre-trained network, from Park et al., 2023. In the expanded Methods we have now given a detailed account on the colour normalisation, Gaussian smoothing, and mini-batch K-means clustering used to isolate initial collagen regions, and the artefact-suppression step that reduces false positives at tissue boundaries before segmentation. These additions provide a more transparent and reproducible description of the pipeline.

The ML code is now uploaded on a public GitHub repository, (reference link - https://github.com/Amaranth-Medical-Analytics/trichrome-collagen) and the link is now provided in our 'Data Availability' statement.

Comment 2. The study is based on an extremely limited sample size (two IDC cases and one benign case), which is insufficient for generalizable conclusions and is more consistent with **a proof-of-concept** than a clinical study.

Response: We agree with the reviewer's valid observation. The scope of our study is methodological feasibility, and to prevent misinterpretation we have revised the manuscript throughout to frame the work explicitly as a proof-of-concept.

In the Abstract we now state that "these findings demonstrate a proof-of-concept, that computational analysis of routine histopathological images can capture key spatial features of collagen…" (Abstract; page no.: 3, line no.: 42).

In the Introduction we state that "we aim to provide a proof-of-concept" and that the comparative analysis "aimed to evaluate methodological feasibility", (Introduction; page no.: 5, line no.: 96).

In the Results we clarify that "Because our objective is methodological validation rather than biological inference, the matched ROIs (not the patients) are the unit of comparison", (Results; page no.: 19, line no.: 377).

This reframing accurately contextualises the scope and utility of the work, and the limited sample size is acknowledged explicitly in the 'Discussion' section, (Discussion; page no.: 30, line no.: 600.)

Comment 3. This study lacks external validation. The proposed pipelines are evaluated only on the same dataset used for development, with no independent cohort for testing. Therefore, there is no evidence demonstrating the generalizability or robustness of the approach across different scanners, staining conditions, or patient populations. Stronger validation on larger and independent datasets is necessary to support the clinical relevance of the method.

Response: This is an excellent point that the reviewer raises. We have explicitly acknowledged this limitation in the 'Discussion' section (Discussion; page no.: 30, line no.: 601), stating that "validation in larger patient cohorts will be necessary to confirm the robustness and generalizability of the observed correlations." All samples in this study were processed, imaged, and analysed in a single batch, so batch effects could not be assessed here; this is now stated as a limitation. As a proof-of-concept, the study validates our digital histology pipelines against SHG on matched ROIs. We are separately preparing a follow-up study for validated methodology to a larger breast-cancer cohort to explore the clinical relevance of the quantified collagen metrics.

Comment 4. The Results section is overly descriptive and lacks a clear narrative structure. Although the authors present extensive statistical analyses, the key findings are not sufficiently highlighted, making it difficult for the reader to identify the main contributions of the study.

Response: We have carried out a major structural revision of the 'Results' section to foreground a clear narrative.

1. Statistical tests' names are now stated once in the 'Methods' rather than repeated for each analysis, redundant descriptive sentences have been removed.
2. Each results subsection now opens with its rationale and closes with a take-away statement.

The streamlined presentation focuses on the differential performance of the modalities and guides the reader to our main conclusion: that digital pathology analysis is concordant with SHG image analysis and is also able to capture heterogeneous collagen architecture across different microenvironments.

The revised writing is submitted with track changes for the editors and reviewers to note the edits and the revision.

Minor concerns

Comment 1. Abbreviations should be defined at first mention.

Response: We have reviewed the entire manuscript and ensured that all abbreviations are defined at their first mention, for example invasive ductal carcinoma (IDC) and two-photon excitation fluorescence (TPEF).

Comment 2. The manuscript reports whole slide imaging at "400× magnification". In digital pathology, magnification terminology can be ambiguous, and it is generally more informative to report the objective magnification (e.g., 20×/40×) and/or pixel resolution (μm/pixel).

Response: We agree and have corrected this in the "WSI acquisition of stained slides" subsection (Methods, 'WSI acquisition of stained slides'; page no.: 9, line no.: 161.) The text now states that slides were "imaged with an objective magnification of 40X and resolution of 0.253 μm/pixel by OptraScan using OS-15 brightfield digital scanner."

Response to Reviewer #2

Summary: This manuscript investigates whether collagen architectural features quantified from digitally scanned Masson-Goldner's Trichrome-stained histopathology sections can approximate measurements obtained from Second Harmonic Generation microscopy. Addressing the following major concerns is necessary before the manuscript can be considered for publication.

We thank Reviewer #2 for the rigorous and detailed assessment, which has substantially improved the statistical framing and reproducibility of the manuscript.

Major comments

Comment 1. The sample size is very limited, with only one benign sample and two IDC samples. Multiple ROIs/FOVs do not substitute for independent biological replicates. The authors should temper claims about scalability, robustness, and clinical implementation, or validate the findings in a larger independent cohort.

Response: We agree that multiple ROIs/FOVs do not substitute for biological replicates and that this sample size cannot support generalised biological or clinical claims. We have reframed the study as a proof-of-concept and methodological validation and have tempered claims throughout the manuscript.

The Abstract and Introduction now state that the findings "demonstrate a proof-of-concept" and that the analysis "aimed to evaluate methodological feasibility" (Abstract, page no.: 3, line no.: 42; Introduction, page no.: 5, line no.: 96.)

Results: We have also edited our results by adding "Because our objective is methodological validation rather than biological inference, the matched ROIs (not the patients) are the unit of comparison; the 25 FOVs within each ROI are treated as repeated (technical) measurements and are not interpreted as independent biological replicates.", (Results; page no.: 19, line no.: 377.)

The Discussion states the limitation directly as: "the analysis was performed on a limited number of tissue samples", (Discussion; page no.: 30, line no.: 601) and that "validation in larger patient cohorts will be necessary to confirm the robustness and generalisability of the observed correlations", (Discussion; page no.: 30, line no.: 601.) We add that this is also necessary to "support clinical relevance"(Discussion; page no.: 30, line no.: 602.)

We have also revised definitive language, describing the framework as "potentially scalable" and removing claims of immediate clinical scalability.

Furthermore, we appreciate the reviewer's suggestion to validate these claims in a larger independent cohort. As a follow-up to this initial proof-of-concept, we have recently completed an assessment of these spatial features of collagen deposition using the digital pipeline described here on a larger breast cancer patient cohort to assess the utility of these metrics for prognosis. This assessment is being prepared for an upcoming manuscript assessing the clinical relevance of these findings.

Comment 2. Statistical analysis may involve pseudoreplication, as multiple FOVs/patches from the same few ROIs and samples appear to be treated as independent. The authors should clarify the unit of analysis and account for nesting or reanalyze results at the ROI/sample level.

Response: We sincerely appreciate the reviewer's rigorous assessment of our statistical methodology. We completely agree that pooling ROIs to represent a patient sample and considering them biologically independent would constitute pseudoreplication, if the goal of this study were to make generalized biological claims about breast cancer patients.

To address this concern and prevent misinterpretation of our reports, we now have explicitly clarified our unit of analysis as the ROIs in the revised manuscript, as described below. Our aim is to evaluate whether our digital pipelines are sensitive enough to detect intra-regional heterogeneity, and have rewritten the results section to reflect this point in the following ways:

> In the SHG analysis section: we have added the clarification "Because our objective is methodological validation rather than biological inference, the matched ROIs (not the patients) are the unit of comparison" (Results, SHG analysis; page no.: 19, line no.: 377.)
>
> In the digital analysis section: we state, "6 selected ROIs were treated as the statistical unit of analysis, to evaluate the pipeline's sensitivity to intra-tumoral collagen architecture heterogeneity."
>
> We have also re-analysed the un-pooled comparisons by performing Kruskal-Wallis tests across the individual ROIs without grouping them by patient sample. Following this, we have updated figures 4 and 5 to reflect these changes.

We believe that the revised statistical analysis and revised framing now reflect the methodological scope of our study appropriately and resolves the misinterpretation as pseudoreplication, as a concern raised in this comment.

Comment 3. Lack of Comparison with Pathology-Specific Feature Encoders: The paper utilizes a standard backbone architecture for feature extraction. However, the manuscript lacks a comparison against different feature encoders, particularly those specifically pre-trained on large-scale pathology data. The authors should consider including pathology-specific encoders (RetCCL and CTransPath), which have been shown to be effective for robust pathological image feature extraction.

Response: We thank the reviewer for this insightful suggestion. We agree that encoders like RetCCL and CTransPath have demonstrated strong performance in histopathology tasks involving haematoxylin and eosin (H&E) stained images, particularly for classification and representation learning.

However, our task differs fundamentally in both imaging modality and prediction objective. Specifically, our method focuses on collagen fibre centreline extraction from digital images of trichrome-stained tumour tissue slides or imaged using SHG-microscopy, which emphasizes fine, filamentous structures rather than cellular or tissue-level semantics typically captured in H&E-based feature representations.

RetCCL and CTransPath are primarily trained on large-scale H&E datasets and are optimized for extracting discriminative features related to nuclei, tissue architecture, and disease patterns. These models operate at patch-level semantics, whereas our task requires pixel- level geometric precision. Collagen fibre analysis requires sensitivity to elongated, low-contrast, and highly anisotropic structures, which are not the primary focus of the encoders such as RetCCL and CTransPath. To the best of our knowledge, there are currently no established benchmarks demonstrating their effectiveness for collagen fibre segmentation or centreline extraction tasks.

Moreover, as we have now explicitly detailed in our revised methods section, saying that "our approach leverages a domain-specific U-Net model that was explicitly trained for collagen fibre centreline detection on SHG images (Park et al., 2023). By adapting Trichrome images into an SHG-like representation, we successfully align the input domain with the training distribution of this model, enabling effective transfer without requiring large-scale retraining or annotations. For these reasons, we believe that our domain adapted U-Net remains the most robust and appropriate architectural choice for this specific task.

Comment 4. The clinical claims are overstated. The study lacks outcome data, diagnostic performance testing, inter-laboratory validation, pathologist comparison, or prospective evaluation. Frame the work as **a methodological feasibility study** rather than evidence of clinical readiness.

Response: We agree that, without outcome data, prospective evaluation, or diagnostic-performance testing, claims of clinical readiness are premature. As detailed in our response to Comment 1, we have revised the Abstract, Introduction, Results, and Discussion to present the work as a methodological feasibility study and proof-of-concept, and to temper all claims accordingly.

Comment 5. The manuscript lacks sufficient introduction to prior work in computational pathology. Several existing AI-based studies in pathology (CHIEF, PROGPath, HiCervix, FMDet, Sac-net, PhaseFIT) have not been discussed, and incorporating these related works would better contextualize the significance of the proposed approach.

Response: We agree that situating our study in the broader landscape of AI advancements would provide crucial context for the reader. Acknowledging these studies will contextualise our approach. To address this, we have added the following to the manuscript's discussion section (page no.: 27, line no.: 540): "There have been highly impactful advances in the field of digital pathology following the development of sophisticated foundational models like CHIEF and PROGPath. While these models demonstrate exceptional performance in extracting patch-level semantics and disease patterns for prognostic tasks, the quantitative assessment of extracellular matrix architecture presents a distinct computational challenge. Extracting spatial metrics from collagen fibres requires a focus on pixel-level geometry and sensitivity to anisotropic structures. This necessitates our domain adapted approach utilizing a U-Net model developed by Park and colleagues, 2023." This clarifies how our pipeline differs from these foundational models.

As discussed in our response to comment 3, many of these foundational models represent developments in the discriminative features of disease classification. These operate primarily on patch-level semantics, while our objective requires to approach the classification at the pixel-level, necessitating our domain adapted approach. To address this distinction, we have added above lines to the Discussion.

We have also updated our references to cite these highly relevant studies (Wang et al, 2024; Yuan W, et al., 2025). We very much appreciate the reviewer for helping us improve the contextual framing of our manuscript.

Comment 6. The exclusion of a low-collagen ROI due to poor SHG signal may introduce bias and overestimate performance. Provide objective exclusion criteria, report sensitivity analyses if possible, and discuss performance in low-collagen or noisy regions.

Response: We appreciate the reviewer's concern regarding potential exclusion bias. In response to this, we have reanalysed the correlations by including the excluded ROI to check for possible changes in concordance due to exclusion bias and clarified our objective exclusion criteria.

We found that, when LC ROI 2 was reintroduced into the Spearman correlation analysis, the strong correlations between SHG and our digital pipelines were completely preserved (SHG vs TWOMBLI - Area: $r = 0.6995$, $p < 0.001$; Alignment: $r = 0.6683$, $p < 0.001$; Fibres: $r = 0.2736$, $p = 0.0007$. SHG vs ML - Area: $r = 0.5717$, $p < 0.0001$; Alignment: $r = 0.5225$, $p < 0.0001$; Fibres: $r = 0.2119$, $p = 0.0095$) The coefficients from this analysis have been added to the supplementary data sheet 3.

Since the excluding the ROI does not affect the correlation drastically, we have proceeded to keep it excluded. We have detailed in the results section that LC ROI 2 was excluded from the analysis because the SHG signal suffered a severe drop in signal-to-noise ratio (SNR), making it practically indistinguishable from the noise. To show this objectively, we reference our orientation histogram analysis, figure 6M alongside the exclusion criteria, providing analytical proof that noise completely overpowered the SHG signal peaking at 0°.

Comment 7. Data and code availability should be improved. Quantifications alone are insufficient for reproducibility; raw or representative de-identified images, ROI annotations, preprocessing scripts, TWOMBLI parameters, and ML code should be provided or justified if unavailable.

Response: Yes, we agree that these points were missing from the previous submission and now, we have improved data and code availability as follows:

- Representative de-identified images are provided as ROI annotations, as shown in Supplementary Fig 1; and the raw images are now uploaded as independent images - https://github.com/Amaranth-Medical-Analytics/trichrome-collagen.
- The preprocessing steps are described in the Methods (the SHG Image Analysis and Digital Image Analysis subsections), and the relevant tools are cited (ImageJ, QuPath, the TWOMBLI macro, and OrientationJ); and the TWOMBLI parameters used for quantification are reported in S1 Dataset.
- The machine-learning code will be made available in a public repository to support full reproducibility with the link mentioned in the manuscript (Methods; page no.: 13, line no.: 246, and Data Availability; page no.: 33, line no.: 656.)

Minor comments

Comment 1. Several grammatical/typographical issues (e.g. “statical test,” “corelation,” “SGH” instead of “SHG,” inconsistent capitalization of ImageJ, fibre/fiber, and Trichrome/trichrome).

Response: We apologise for these oversights. We have thoroughly proofread the manuscript, corrected the typographical errors, and standardised spelling and capitalisation (including SHG, ImageJ, fibre, and Trichrome) throughout.

Comment 2. The authors should explain how staining variability, scanner variability, and batch effects were handled, since these are critical issues for any digital pathology-based pipeline.

Response: We agree that mitigating these variables is crucial for a robust digital pathology pipeline. As this study was designed as a proof-of-concept methodological validation, all samples were processed, imaged and analysed together in one batch to minimise processing variability.

To ensure this point is explicit to the reader, we have added the following to “Patient Tissue Samples” in the methods section: “To minimise batch effects all samples were processed in a single batch.” We have also mentioned that it would be necessary to account for “batch effects and staining variability in these pipelines” when discussing the limitations in the discussion section.

Our follow-up studies will focus on utilizing this validated methodology across a larger independent cohort and evaluating the pipeline’s robustness against staining variability.

Response to Reviewer #3

Comments: Overall the article is interesting in theory comparing new methods between each other as well as to gold standards. There are some major and minor comments that need to be addressed as follows:

We thank Reviewer #3 for the detailed, line-by-line reading, which has improved the clarity and accuracy of the manuscript.

Major and minor comments

Comment 1. page 9 line 179 discusses multiple images. Why different imaging region sizes and number of images?

Response: We have added the rationale for each imaging round to the "Experimental setup for SHG…" subsection of the Methods (page no.: 9, line no.: 177). The updated text reads as follows: "In the first round of imaging, three 100 × 100 μm^2 FOVs were captured referencing the ROI annotations for the IDC High Collagen sample to specifically validate the SHG signal and assess compatibility across different staining protocols (S1 Fig). Once confirmed that SHG microscopy can be done on Trichrome-stained slides to visualise collagen fibres, we carried out a sound round of imaging to capture a larger tissue area to visualise and quantify macroscopic collagen architecture. Digitally scanned images span across the entire tissue sample, whereas SHG image acquisition of such a large region would require a significantly large amount of time. Therefore, in the second round of imaging, 25 images each of size 103 × 103 μm^2 (234 × 234 $pixels^2$) of 0.44 µm pixel size with an overlap region of 11 µm were acquired and stitched to generate a ROI of size 470 × 470 μm^2 compromising on time taken to image while also capturing a larger tissue region."

Comment 2. *Page 9 line 188 mentions FOV as 110 um, is this a typo or a discrepancy?*

Response: The value 110 is not a typo: it accounted for a 10 µm overlap region required for stitching. During revision, however, we re-verified the raw SHG files and calibration slides and found that the originally reported dimensions were approximations. The exact raw SHG FOV is 234 pixels (~103 µm) with a 25-pixel (~11 µm) overlap. We have corrected these values consistently throughout the manuscript.

In addition, we have now added supplementary figure 2 (S2 Fig), which details the USAF calibration of the SHG microscope to get the SHG pixel size, along with the original pixel dimensions of the FOV, overlap and stitched ROI.

Comment 3. *Page 10 line 193, images are binarized. Explain why this was done for clarity. Is it to make it black/white?*

Response: Binarization is a segmentation step that enforces a strict separation between fibre signal and background; the visual result is indeed a black-and-white image. We have updated the "SHG Image Analysis" subsection to state that "to ensure strict separation between fibre and background, binarization was done…" resulting in "white fibres against a black background." (Methods, 'SHG Image Analysis'; page no.: 11, line no.: 207.)

Comment 4. page 10 line 95 "OR" doesn't need to be capitalized

Response: The capitalisation was intentional to denote the Boolean logical operator used in the analysis pipeline. For clarity we have updated the methods to place the operator in quotation marks as "OR operation."

Comment 5. page 10 line 202 regarding fiber width: Fiber width is hard to estimate across different patients let alone tumor types. Given that a cut is essentially a 2D structure, how can you ensure you're assessing fiber widths that are comparable across the different samples

Response: We agree that estimating fibre width from a 2D section is challenging due to sectioning artefacts. However, because this is a methodological comparison using matched ROIs on the same tissue slice, these limitations apply equally to all ROIs and both pipelines, so the cross-modality comparisons remain valid; stitching multiple FOVs into larger ROIs further averages out local sectioning artefacts. We have now added this as an inherent limitation of 2D digital pathology in the Discussion, noting that the effect is minimised when imaging larger tissue areas, (Discussion; page no.: 31, line no.: 620.)

Comment 6. page 10 line 204 for percentage of collagen area from binary image: Can this suppress/remove other cell types and thus artificially inflating collagen %?

Response: We would like to point out that since the percentage is calculated with respect to the total fixed area of the image patch, supressing the non-collagenous components does not inflate the collagen area. By removing the non-collagenous regions during deconvolution and binarization, we simply ensure they are excluded from the being counted as collagen pixels. To ensure this is explicit we have revised the text to state that the percentage was calculated through "the ratio of collagen-positive pixels with respect to the total pixel area of the image" (Methods, 'SHG Image Analysis'; page no.: 11, line no.: 223.)

We thank the reviewer for helping us to clarify this detail.

Comment 7. (p10, l209) Is TWOMBLI widely used? If so, state as such.

Response: Yes. We have added that the TWOMBLI macro is "a widely used tool specifically developed to quantify extracellular matrix patterns," highlighting its established utility, (Methods; page no.: 12, line no.: 234)

Comment 8. page 11 line 224, has "(citation)", missing?

Response: Thank you for catching this. The missing citation has been added. Citation - Püspöki Z, Storath M, Sage D, Unser M. Transforms and Operators for Directional Bioimage Analysis: A Survey. In: De Vos WH, Munck S, Timmermans JP, editors. Focus on Bio-Image Informatics [Internet]. Cham: Springer International Publishing; 2016 [cited 2026 Feb 13]. page no.: 69–93. (Advances in Anatomy, Embryology and Cell Biology). Available from: http://link.springer.com/10.1007/978-3-319-28549-8_3 doi:10.1007/978-3-319-28549-8_3.

Comment 9. *Page 11 line 227 for the multistep approach, this should be briefly explained*

Response: We thank the reviewer for highlighting the need for more methodological detail in this section. As the reviewer noted, in alignment with similar feedback from reviewer #1, the description of our ML

pipeline required elaboration, and now it has been added (Methods; page no.: 13, line no.: 247,) as follows:

We have expanded the "Digital Analysis with ML" subsection of the methods to explicitly outline the multistep approach. We have detailed: domain adaptation, how brightfield images were transformed into 'SHG-like' using colour normalisation and the green channel as the primary surrogate; clustering parameter and the use of Gaussian smoothing followed by Mini-Batch K-Means clustering; as well as artefact suppression, where the residual tissue mask was dilated and subtracted prior to the U-Net segmentation.

We believe this comprehensive rewrite provides the required computational transparency. We sincerely thank the reviewer for prompting this necessary elaboration.

Comment 10. *page 11 line 229, dynamic background detection logic should be explained, even briefly*

Response: We have updated the "Digital Image Analysis with ML" subsection to define this logic: background clusters are identified iteratively using high-intensity thresholds, and the remaining clusters are classified as collagen based on green-channel dominance and non-grey colour variation. This ensures that faint fibres are detected while minimising false positives. The detailed explanation is now added to the Methods section; page no.: 13, line no.: 257.

Comment 11. *page 13 line 264-269 from "three sections were of 3 to 5 um..." shoud this be in methods?*

Response: We agree. This sentence has been removed from the Results to reduce redundancy, as it is already described in the Methods.

Comment 12. page 13 line 273 for combining image of shg and tpef, why combined?

Response: The SHG and TPEF channels were combined to visualise fibrillar collagen (SHG) alongside surrounding cellular features (TPEF) within the same field of view. The Results now clarify that overlaying the signals highlights fibrillar collagen in the context of the tissue's cellular structures.

Comment 13. page 14 line 293 for unstained slides, why included in the first place?

Response: Unstained sections represent the conventional, gold-standard practice for SHG imaging. We included them as a baseline to check for any effect of staining on SHG imaging, since a goal of the study was to perform SHG on Trichrome-stained slides. We have added a phrase in the Results to make this rationale explicit.

Comment 14. page 14 line 295, for FOV 110x110 (discrepent from previously mentioned 100) stiched, this should be explained earlier maybe in methods?

Response: As in our response to Comment 2, we re-verified the raw data and updated the dimensions to the exact measurements. The 25 FOVs (each ~103 × 103 µm²) are stitched with an ~11 µm overlap to generate a final ROI of ~470 × 470 µm² (previously approximated as 500 µm). This is now stated clearly in both the Methods (page no.: 10, line no.: 184) and Results (page no.: 14, line no.: 331.)

The exact dimensions can also be found in the revised manuscript, supplementary figure 2.

Comment 15. page 15 line 313, why difference in size of 500 or 1150 to 400 or 1716?

Response: This observation led us to identify and correct a resolution error. We have referred to the USAF Calibration of the SHG microscope to determine the pixel size. We find that the SHG microscope acquires at 0.44 µm/pixel while the brightfield scanner acquires at a different resolution, so the ROI pixel dimensions differ between modalities. On rechecking, the raw SHG FOVs are 234 pixels with a 25-pixel overlap, giving a stitched ROI of ~1077 pixels. We also found a metadata-encoding error in the scanner's proprietary software, which displayed a default resolution of 0.233 µm/pixel; the true resolution is 0.253 µm/pixel. Using these corrected values, the SHG ROI is ~470 × 470 µm² (1077 × 1077 pixels) and the corresponding digital ROI is ~435 × 435 µm² (1716 × 1716 pixels). As previously noted in the limitations, differences in spatial resolution required approximate rather than exact pixel-level ROI matching. The text has been updated throughout to reflect these corrected values in the Methods (page no.: 10, line no.: 184 and line no.:195; page no.: 11, line no.: 223.)

The USAF calibration is added to the new supplementary figure S2, and the metrics of the same have been included in the supplementary data sheet 1.

Comment 16. page 15 line 316, why only 2 ROIs? who chose the ROIs?

Response: The ROIs were annotated by a senior author (MK) to be representative of the spatial architectures of each tissue type. SHG imaging was limited to two large, stitched ROIs per sample because SHG acquisition is highly time-intensive, and these regions were taken as sufficient for a proof-of-concept study. The Methods now state that the ROIs were annotated by MK based on representative architecture.

Comment 17. page 17 line 343, why average SHG signal values and not another weighted method to ensure unbiasing of skews? especially considering the results from line 344 to 347

Response: The average SHG signal is an established and robust metric for comparing regions of differing collagen density. Because our pipeline excludes non-collagenous background pixels through binarization, the average is computed strictly over segmented fibril pixels, so it is not skewed by empty space; SHG signal strength is positively correlated with collagen fibrillar density, as established in prior work (Jyothsna et al., 2021). We have added a clarification and this citation to the "SHG Image Analysis" subsection, which reads as follows, "as background pixels are completely excluded by the mask, the average intensity provides an unbiased metric that is positively correlated with collagen fibrillar density" (Methods; page no.: 11, line no.: 205.

Comment 18. page 18 line 386, "although not significant", this isn't statistically signicant but could be still valuable trend to mention.

Response: We have re-evaluated the analysis treating the 6 ROIs as the unit of analysis rather than grouping by sample type. In the revised ROI-to-ROI comparisons this comparison now reaches statistical significance, and the relevant text has been rewritten accordingly in the results (page no.: 21, line no.: 412 and line no.: 415.)

Comment 19. page 23 line 497 regarding accessibility, what resources would this require for on prem deployment? this might be restricted to high end computers?

Response: We appreciate the reviewers practical query. We are pleased to clarify our pipeline is not restricted to high-end computers. All components of the pipeline can be executed on a standard computer. In our implementation, the pipeline runs on a system with 8 CPU threads and 32 GB RAM. We adopt a tile-based processing strategy that prevents loading the entire WSI at once, which combined with multiprocessing across CPU threads achieves high throughput while maintaining controlled resource usage.

We have updated our manuscript's results section to highlight moderate hardware requirements and our processing strategy (Results; page no.: 25, line no.: 504)

Comment 20. (p24) The table has odd word spacing.

Response: We have corrected this formatting artefact and reduced the line spacing to make the table more compact and readable.

Comment 21. p 26 line 535, why not normalize pixel sizes between SHG image and digital image?

Response: We deliberately did not normalise pixel sizes, because down-sampling the higher-resolution digital image to match the SHG resolution would blur it and lose information. The comparison techniques used here are agnostic to pixel size, as they extract collagen-specific features and signal strength across fibres that span many pixels. This is in fact an advantage of the approach: it allows comparison across images acquired with different modalities and field sizes without resampling. We have clarified this in the discussion (page no.: 28, line no.: 559).

Comment 22. (p28, l568) Regarding routine clinical workflows — how does this matter to oncologists, and how would pathologists deploy it in practice?

Response: We thank the reviewer for prompting us to clarify the practical implications of our work. The present study is designed specifically as methodological proof-of-concept, we do not draw any clinical or

prognostic inferences. However, through validation of the pipelines that are tested and proposed here in this study, on routinely stained slides, we lay the groundwork for future clinical utility.

This has been clarified in the discussion (page no.: 30, line no.: 601.), as follows:

We are preparing an upcoming manuscript as a follow-up to this initial proof-of-concept, where we assess the spatial features of collagen deposition using the digital pipeline described here on a larger breast cancer patient cohort to investigate the utility of these metrics for prognosis.

Comment 23. (p28) regarding limitations, Every institution has its own staining protocol. how would this effect the results? This is a limitation that should be addressed.

Response: Both pipelines have flexibility to handle staining heterogeneity: the ImageJ/QuPath pipeline allows custom colour-deconvolution vectors matched to specific stain intensities, and the ML pipeline includes a colour-normalisation step designed to minimise staining variability. We nonetheless agree this is a limitation and now state in the discussion (page no.: 31, line no.: 610) that "validation across multi-institutional cohorts would be necessary to confirm robustness of these tools against diverse real-world staining practices."

Comment 24. The text could be minimized significantly; there are many unnecessary redundancies.

Response: We have removed the redundant summary paragraph from the Introduction, deleted repeated technique descriptions from the Results, and edited heavily throughout. These changes tighten the narrative and improve readability.

Typographical comments. Various typos: (l311) "further to" vs "to further"; (l334) "*-" typo; (l420) "second harmonic generation" spelled out could be abbreviated; (l440) typo; (l445) should be a comma; (l503) SHG written in full; random word/phrase bolding.

Response: We thank the reviewer for their careful reading of our manuscript and apologise for these oversights. We have thoroughly proofread the revised manuscript to ensure maximum no typographical and formatting errors.

We thank the editors and all three reviewers again for their time and constructive feedback, which have substantially strengthened the manuscript. We hope the revised version is now suitable for publication in PLOS Digital Health.

On behalf of all authors,

Dr. Madhura Kulkarni (Corresponding Author)
