# Supplementary material for: Quantitative assessment of collagen architecture from routine histopathological images shows concordance with Second Harmonic Generation microscopy
Source: PLOS Digit Health. 2026 Oct 26;5(10):e0001364. doi: 10.1371/journal.pdig.0001364 (PMC13704883; doi:10.1371/journal.pdig.0001364)
Supplement: Peer review history

# Peer review: Decision Letter 0

**DOI:** 10.1371/journal.pdig.0001364.r001

**Authors:** Feng Liu, Junhan Zhao

11 Jun 2026

Response to Reviewers Revised Manuscript with Track Changes Manuscript **Journal Requirements:**

1. Thank you for stating the following in the Financial Disclosure section:

[DBT Ramalingaswami ‘re-entry’ fellowship awarded by DBT India to MK.

https://rcb.res.in/RRF/

Ashoka University and Mphasis AI and Applied Tech Labs funding the TNBC – CHART project for LSS.

https://www.ashoka.edu.in/page/mphasis-lab/

Bajaj Pvt Ltd for CSR funding to PCCM.

https://www.bajajauto.com/corporate/corporate-social-responsibility

The funders had no role in study design, data collection and analysis, decision to publish, or preparation of the manuscript.].

We note that you received funding from a commercial source: [Bajaj Pvt Ltd]

Please provide an amended Competing Interests Statement that explicitly states this commercial funder, along with any other relevant declarations relating to employment, consultancy, patents, products in development, marketed products, etc.

Within this Competing Interests Statement, please confirm that this does not alter your adherence to all PLOS policies on sharing data and materials by including the following statement: ""This does not alter our adherence to PLOS policies on sharing data and materials.” (as detailed online in our guide for authors http://journals.plos.org/plosone/s/competing-interests).  If there are restrictions on sharing of data and/or materials, please state these. Please note that we cannot proceed with consideration of your article until this information has been declared.

Please update your Competing Interests Statement within the submission form upon resubmission of your manuscript. If you are unable to do so, please email the journal office, and we may make these updates on your behalf.

2. Some material included in your submission may be copyrighted. According to PLOS’s copyright policy, authors who use figures or other material (e.g., graphics, clipart, maps) from another author or copyright holder must demonstrate or obtain permission to publish this material under the Creative Commons Attribution 4.0 International (CC BY 4.0) License used by PLOS journals. Please closely review the details of PLOS’s copyright requirements here: PLOS Licenses and Copyright. If you need to request permissions from a copyright holder, you may use PLOS's Copyright Content Permission form.

Please respond directly to this email or email the journal office and provide any known details concerning your material's license terms and permissions required for reuse, even if you have not yet obtained copyright permissions or are unsure of your material's copyright compatibility.

Potential Copyright Issues:

a. Figures 1, 2, 3, 4, 5, 6, 7, S1 Fig: Please confirm whether you drew the images / clip-art within the figure panels by hand. If you did not draw the images, please provide (a) a link to the source of the images or icons and their license / terms of use; or (b) written permission from the copyright holder to publish the images or icons under our CC-BY 4.0 license. Alternatively, you may replace the images with open source alternatives. See these open source resources you may use to replace images / clip-art:

- https://commons.wikimedia.org

- https://openclipart.org/

b. We do not publish any copyright or trademark symbols that usually accompany proprietary names, eg (R), (C), or TM  (e.g. next to drug or reagent names). Please remove all instances of trademark/copyright symbols throughout the text, including PathPresenter®.

3. We ask that a manuscript source file is provided at Revision. Please upload your manuscript file as a .doc, .docx, .rtf or .tex.

**Additional Editor Comments (if provided):** **Reviewers' Comments:**

**Comments to the Author**

1. Does this manuscript meet PLOS Digital Health’s publication criteria?

Reviewer #1: Partly

Reviewer #2: Yes

Reviewer #3: Yes

**********

2. Has the statistical analysis been performed appropriately and rigorously?

Reviewer #1: Yes

Reviewer #2: Yes

Reviewer #3: Yes

**********

3. Have the authors made all data underlying the findings in their manuscript fully available (please refer to the Data Availability Statement at the start of the manuscript PDF file)?

The PLOS Data policy

Reviewer #1: No

Reviewer #2: No

Reviewer #3: Yes

**********

4. Is the manuscript presented in an intelligible fashion and written in standard English?

Reviewer #1: Yes

Reviewer #2: Yes

Reviewer #3: Yes

**********

**Reviewer #1:**  Summary

This study explores whether collagen structural features derived from routine histopathological images can approximate those obtained from SHG microscopy, which addresses a problem in digital pathology. However, the novelty is moderate, since similar approaches for collagen quantification from histology have already been reported. The main contribution of this work is the cross-modality validation, rather than proposing a new methodology. The study is generally well designed, especially in terms of multi-pipeline comparison and ROI matching. Nevertheless, it is limited by a very small sample size and the lack of external validation. In addition, the machine learning part needs more detailed description and more comprehensive evaluation. This work is promising, but stronger validation is still needed to support its clinical applicability.

Major Concerns

1. The machine learning pipeline using U-Net combined with clustering is not described in sufficient detail. Several important aspects are missing, including the size of the training dataset, the definition and source of ground truth annotations, the validation strategy, and the performance metrics used such as Dice coefficient and IoU. Without this information, it is difficult to evaluate the reliability and robustness of the model. In addition, the current description does not allow the study to be reproduced. A more complete and transparent description of the machine learning methodology is necessary, and providing the code on GitHub would improve reproducibility.

2. The study is based on an extremely limited sample size, including only two IDC cases and one benign case. This is insufficient to support any generalizable conclusions and is more consistent with a proof-of-concept rather than a clinical study.

3. This study lacks external validation. The proposed pipelines are evaluated only on the same dataset used for development, with no independent cohort for testing. Therefore, there is no evidence demonstrating the generalizability or robustness of the approach across different scanners, staining conditions, or patient populations. Stronger validation on larger and independent datasets is necessary to support the clinical relevance of the method.

4. The Results section is overly descriptive and lacks a clear narrative structure. Although the authors present extensive statistical analyses, the key findings are not sufficiently highlighted, making it difficult for the reader to identify the main contributions of the study.

Minor Concerns

1. Abbreviations should be defined at first mention.

2. The manuscript reports whole slide imaging at “400× magnification”. In digital pathology, magnification terminology can be ambiguous, and it is generally more informative to report the objective magnification (e.g., 20×/40×) and/or pixel resolution (μm/pixel).

**Reviewer #2:**  This manuscript investigates whether collagen architectural features quantified from digitally scanned Masson-Goldner’s Trichrome-stained histopathology sections can approximate measurements obtained from Second Harmonic Generation microscopy. Addressing the following major concerns is necessary before the manuscript can be considered for publication.

Major Comments

1. The sample size is very limited, with only one benign sample and two IDC samples. Multiple ROIs/FOVs do not substitute for independent biological replicates. The authors should temper claims about scalability, robustness, and clinical implementation, or validate the findings in a larger independent cohort.

2. Statistical analysis may involve pseudoreplication, as multiple FOVs/patches from the same few ROIs and samples appear to be treated as independent. The authors should clarify the unit of analysis and account for nesting or reanalyze results at the ROI/sample level.

3. Lack of Comparison with Pathology-Specific Feature Encoders: The paper utilizes a standard backbone architecture for feature extraction. However, the manuscript lacks a comparison against different feature encoders, particularly those specifically pre-trained on large-scale pathology data. The authors should consider including pathology-specific encoders (RetCCL and CTransPath), which have been shown to be effective for robust pathological image feature extraction.

4. The clinical claims are overstated. The study lacks outcome data, diagnostic performance testing, inter-laboratory validation, pathologist comparison, or prospective evaluation. The authors should frame the work as a methodological feasibility study rather than evidence of clinical readiness.

5. The manuscript lacks sufficient introduction to prior work in computational pathology. Several existing AI-based studies in pathology (CHIEF, PROGPath, HiCervix, FMDet, Sac-net, PhaseFIT) have not been discussed, and incorporating these related works would better contextualize the significance of the proposed approach.

6. The exclusion of a low-collagen ROI due to poor SHG signal may introduce bias and overestimate performance. The authors should provide objective exclusion criteria, report sensitivity analyses if possible, and discuss performance in low-collagen or noisy regions.

7. Data and code availability should be improved. Quantifications alone are insufficient for reproducibility; raw or representative de-identified images, ROI annotations, preprocessing scripts, TWOMBLI parameters, and ML code should be provided or justified if unavailable.

Minor Comments

1. Several grammatical and typographical issues should be corrected. Examples include “statical test,” “corelation,” “SGH” instead of “SHG,” and inconsistent capitalization of ImageJ, fibre/fiber, and Trichrome/trichrome.

2. The authors should explain how staining variability, scanner variability, and batch effects were handled, since these are critical issues for any digital pathology-based pipeline.

**Reviewer #3:**  Overall the article is interesting in theory comparing new methods between each other as well as to gold standards. There are some major and minor comments that need to be addressed as followes:

1. page 9 line 179 discusses multiple images. Why different imaging region sizes and number of images?

2. Page 9 line 188 mentions FOV as 110 um, is this a typo or a discrepency?

3. Page 10 line 193, images are binarized. Explain why this was done for clarity. Is it to make it black/white?

4. page 10 line 95 "OR" doesn't need to be capitalized

5. page 10 line 202 regarding fiber width: Fiber width is hard to estimate across different patients let alone tumor types. Given that a cut is essentially a 2D structure, how can you ensure you’re assessing fiber widths that are comparable across the different samples

6. page 10 line 204 for precentage of collagen area from binary image: Can this suppress/remove other cell types and thus artifically inflating collagen %?

7. page 10 line 209, TWOMBLI, Is this widely used? If so, a quick sentence stating as such

8. page 11 line 224, has "(citation)", missing?

9. Page 11 line 227 for the multistep approach, this should be briefly explained

10. page 11 line 229, dynamic background detection logic should be explained, even briefly

11. page 13 line 264-269 from "three sections were of 3 to 5 um..." shoud this be in methods?

12. page 13 line 273 for combining image of shg and tpef, why combined?

13. page 14 line 293 for unstained slides, why included in the first place?

14. page 14 line 295, for FOV 110x110 (discrepent from previously mentioned 100) stiched, this should be explained earlier maybe in methods?

15. page 15 line 313, why difference in size of 500 or 1150 to 400 or 1716?

16. page 15 line 316, why only 2 ROIs? who chose the ROIs?

17. page 17 line 343, why average SHG signal values and not another weighted method to ensure unbiasing of skews? especially considering the results from line 344 to 347

18. page 18 line 386, "although not significant", this isn't statistically signicant but could be still valuable trend to mention

19. page 23 line 497 regarding accessibility, what resources would this require for on prem deployment? this might be restricted to high end computers?

20. page 24, table has odd word spacing

21. p 26 line 535, why not normalize pixel sizes between SHG image and digital image?

22. p 28 568, regarding routine clinical workflows, How does this matter to oncologists and how do pathologists deploy this in practice

23. p 28 regarding limitations, Every insitution has its own staining protocol. how would this effect the results?

This is a limitation that should be addressed

24. the text could be minimized signicantly. There is a lot of redundancies that are unncessary

Many typographical errors like page 15 line 311 "further to" rather than 'to further', page 16 line 334 "*-" typo, line 420 "second harmonic generation" is written out in full but could be truncated, line 440 typo, line 445 should be comma, line 503 shg fully written, random word/phrase bolding,

**********

what does this mean?). If published, this will include your full peer review and any attached files.

**Do you want your identity to be public for this peer review?**  If you choose “no”, your identity will remain anonymous but your review may still be made public.

For information about this choice, including consent withdrawal, please see our Privacy Policy

Reviewer #1: No

Reviewer #2: No

Reviewer #3: No

**********

**Figure resubmission:**

**Reproducibility:**

To enhance the reproducibility of your results, we recommend that authors of applicable studies deposit laboratory protocols in protocols.io, where a protocol can be assigned its own identifier (DOI) such that it can be cited independently in the future. Additionally, PLOS ONE offers an option to publish peer-reviewed clinical study protocols. Read more information on sharing protocols at https://plos.org/protocols?utm_medium=editorial-email&utm_source=authorletters&utm_campaign=protocols

# Author comment: Author response to Decision Letter 1

**DOI:** 10.1371/journal.pdig.0001364.r002

9 Aug 2026

Attachment Submitted filename: Response_to_Reviewers_Ingawale-et-al-2026.pdf

# Peer review: Decision Letter 1

**DOI:** 10.1371/journal.pdig.0001364.r003

**Authors:** Feng Liu, Junhan Zhao

7 Oct 2026

Quantitative assessment of collagen architecture from routine histopathological images shows concordance with Second Harmonic Generation microscopy

PDIG-D-26-00512R1

Dear Dr. Kulkarni,

We are pleased to inform you that your manuscript 'Quantitative assessment of collagen architecture from routine histopathological images shows concordance with Second Harmonic Generation microscopy' has been provisionally accepted for publication in PLOS Digital Health.

Before your manuscript can be formally accepted you will need to complete some formatting changes, which you will receive in a follow-up email from a member of our team.

Please note that your manuscript will not be scheduled for publication until you have made the required changes, so a swift response is appreciated.

IMPORTANT: The editorial review process is now complete. PLOS will only permit corrections to spelling, formatting or significant scientific errors from this point onwards. Requests for major changes, or any which affect the scientific understanding of your work, will cause delays to the publication date of your manuscript.

If your institution or institutions have a press office, please notify them about your upcoming paper to help maximize its impact. If they'll be preparing press materials, please inform our press team as soon as possible -- no later than 48 hours after receiving the formal acceptance. Your manuscript will remain under strict press embargo until 2 pm Eastern Time on the date of publication. For more information, please contact digitalhealth@plos.org.

Thank you again for supporting Open Access publishing; we are looking forward to publishing your work in PLOS Digital Health.

Best regards,

Junhan Zhao

Academic Editor

PLOS Digital Health

***********************************************************

**Additional Editor Comments (if provided):**

All the reviewers are satisfied with the revised work. An acceptance is suggested.

**Reviewer Comments (if any, and for reference):**

Reviewer's Responses to Questions

**Comments to the Author**

Reviewer #1: All comments have been addressed

Reviewer #2: (No Response)

**********

publication criteria?

Reviewer #1: Yes

Reviewer #2: (No Response)

**********

3. Has the statistical analysis been performed appropriately and rigorously?

Reviewer #1: Yes

Reviewer #2: (No Response)

**********

4. Have the authors made all data underlying the findings in their manuscript fully available (please refer to the Data Availability Statement at the start of the manuscript PDF file)?

The PLOS Data policy

Reviewer #1: Yes

Reviewer #2: (No Response)

**********

5. Is the manuscript presented in an intelligible fashion and written in standard English?

Reviewer #1: Yes

Reviewer #2: (No Response)

**********

**Reviewer #1:**  The authors have improved the manuscript and addressed most of my previous concerns. The manuscript is now more appropriately presented as a methodological proof-of-concept, which better matches the limited number of biological samples and the lack of external validation.

One minor concern. Although the U-Net was trained and validated by Park’s paper, this study adds a domain-adaptation step that converts Trichrome images into an SHG-like representation before applying the pre-trained model. Therefore, the previously reported performance of the U-Net does not directly validate the full pipeline used in this study. I would support publication after minor clarification of the point above.

**Reviewer #2:**  (No Response)

**********

what does this mean?). If published, this will include your full peer review and any attached files.

**Do you want your identity to be public for this peer review?**  If you choose “no”, your identity will remain anonymous but your review may still be made public.

For information about this choice, including consent withdrawal, please see our Privacy Policy

Reviewer #1: No

Reviewer #2: None

**********
